# Oncofusion-driven *de novo* enhancer assembly promotes malignancy in Ewing sarcoma via aberrant expression of the stereociliary protein LOXHD1

**DOI:** 10.1016/j.celrep.2022.110971

**Published:** 2022-06-14

**Authors:** Qu Deng, Ramakrishnan Natesan, Florencia Cidre-Aranaz, Shehbeel Arif, Ying Liu, Reyaz ur Rasool, Pei Wang, Erick Mitchell-Velasquez, Chandan Kanta Das, Endrit Vinca, Zvi Cramer, Patrick J. Grohar, Margaret Chou, Chandan Kumar-Sinha, Kristy Weber, T.S. Karin Eisinger-Mathason, Nicolas Grillet, Thomas Grünewald, Irfan A. Asangani

**Affiliations:** 1 Department of Cancer Biology, Perelman School of Medicine, University of Pennsylvania, 421 Curie Boulevard, BRBII/III, Philadelphia, PA 19104, USA; 2 Max-Eder Research Group of Pediatric Sarcoma Biology, Institute of Pathology, LMU Munich, Munich, Germany; 3 Hopp Children’s Cancer Center (KiTZ) Heidelberg, Heidelberg, Germany; 4 Division of Translational Pediatric Sarcoma Research, German Cancer Research Center (DKFZ), German Cancer Consortium (DKTK), Hopp Children’s Cancer Center (KiTZ), Institute of Pathology, Heidelberg University Hospital, Heidelberg, Germany; 5 Institute of Pathology, Heidelberg University Hospital, Heidelberg, Germany; 6 Department of Pathology and Laboratory Medicine, Perelman School of Medicine, University of Pennsylvania, BRBII/III, Philadelphia, PA, USA; 7 Department of Otolaryngology-Head & Neck Surgery, School of Medicine, Stanford University, Stanford, CA, USA; 8 Children’s Hospital of Philadelphia, Philadelphia, PA, USA; 9 Michigan Center for Translational Pathology, University of Michigan, Ann Arbor, MI, USA; 10 Department of Orthopaedic Surgery, Perelman School of Medicine, University of Pennsylvania, Philadelphia, PA, USA; 11 Abramson Family Cancer Research Institute, Perelman School of Medicine, University of Pennsylvania, Philadelphia, PA, USA; 12 Epigenetics Institute, Perelman School of Medicine, University of Pennsylvania, Philadelphia, PA, USA; 13 These authors contributed equally; 14 Lead contact

## Abstract

Ewing sarcoma (EwS) is a highly aggressive tumor of bone and soft tissues that mostly affects children and adolescents. The pathognomonic oncofusion EWSR1::FLI1 transcription factor drives EwS by orchestrating an oncogenic transcription program through *de novo* enhancers. By integrative analysis of thousands of transcriptomes representing pan-cancer cell lines, primary cancers, metastasis, and normal tissues, we identify a 32-gene signature (ESS32 [Ewing Sarcoma Specific 32]) that stratifies EwS from pan-cancer. Among the ESS32, LOXHD1, encoding a stereociliary protein, is the most highly expressed gene through an alternative transcription start site. Deletion or silencing of EWSR1::FLI1 bound upstream *de novo* enhancer results in loss of the LOXHD1 short isoform, altering EWSR1::FLI1 and HIF1α pathway genes and resulting in decreased proliferation/invasion of EwS cells. These observations implicate LOXHD1 as a biomarker and a determinant of EwS metastasis and suggest new avenues for developing LOXHD1-targeted drugs or cellular therapies for this deadly disease.

## In brief

Deng et al. use an integrative bioinformatics analysis of multiple transcriptomic datasets and functional assays to identify LOXHD1 as a highly specific EWSR1::FLI1 target in EwS. LOXHD1 silencing results in attenuated hypoxic response and tumorigenicity. These results implicate LOXHD1 as a biomarker and a potential therapeutic target in EwS.

## Graphical Abstract

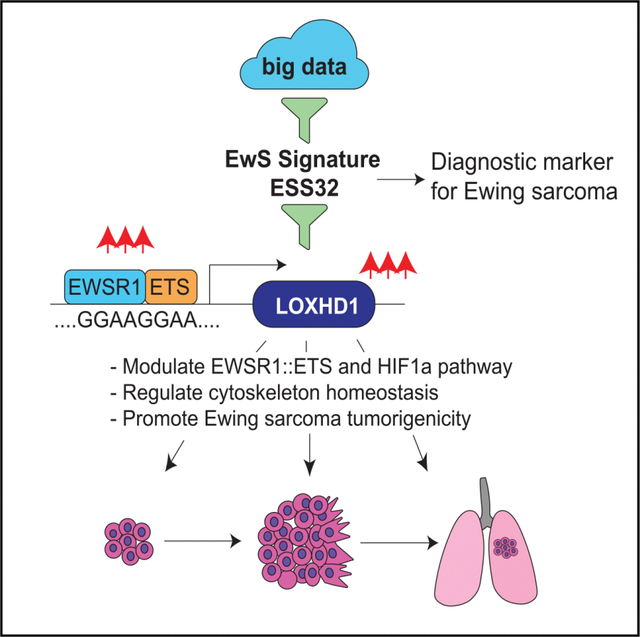

## INTRODUCTION

Ewing sarcoma (EwS) is the second most common malignant bone or soft tissue cancer predominantly affecting children and young adults (Grünewald et al., 2018). Although the 5-year survival rate for primary EwS initially improved following the introduction of systemic chemotherapy in the neoadjuvant and adjuvant setting, several clinical studies indicate a plateau phase for these conventional therapies (Stahl et al., 2011). Further, the prognosis for patients with high-risk recurrent disease is abysmal with <10% survival at 5 years; therefore, novel therapies are urgently needed to improve outcomes (Esiashvili et al., 2008; Gaspar et al., 2015; Grünewald et al., 2018). EwS is driven by chromosomal translocations that generate pathognomonic fusions between the EWSR1 gene with variable members of the ETS family of transcription factors, most commonly FLI1 (85% of cases) (Delattre et al., 1992; Sorensen et al., 1994). In the remaining 15% to 20% of EwS, variant fusions between EWSR1 and other members of the ETS family occur, most commonly ERG (Sorensen et al., 1994).

EWSR1::FLI1 functions as a pioneer transcription factor by preferentially binding to genomic regions enriched for polymorphic GGAA microsatellites and induces chromatin reorganization resulting in the formation of opportunistic *de novo* enhancers and super-enhancers (Gangwal et al., 2008; Riggi et al., 2014). Specifically, EWSR1::FLI1 binding to GGAA microsatellite repeats leads to the recruitment of the BRG1–BRM-associated factor (BAF) chromatin-remodeling complex (Boulay et al., 2017), BRD4 chromatin readers (Gollavilli et al., 2018), lysine-specific demethylase (LSD1) (Theisen et al., 2020), and RNA PolII (Kwon et al., 2013), resulting in the establishment of *de novo* enhancers and activation of EwS transcriptional program (Grünewald et al., 2018; Pishas and Lessnick, 2016). Although EWSR1::FLI1 would in principle constitute the most obvious and highly specific therapeutic target; this oncofusion protein represents a drug discovery challenge, because of its activity as targeting transcription factor and unstructured prion-like domains in the EWSR1 portion of the fusion (Boulay et al., 2017; Knott et al., 2019). Given the paucity of druggable targets, the identification of novel Ewing-specific oncogenes and mediators of EWSR1::FLI1 are urgently required. While cytogenetic evaluation of pathognomonic fusion is used to diagnose EwS, highly specific EWSR1::ETS-driven gene/gene signatures are needed to confirm the diagnosis and progression of the disease.

Here, we use an integrative RNA-sequencing (RNA-seq)-based approach, coupled with chromatin immunoprecipitation sequencing (ChIP-seq) and tumor cell functional studies, to identify stereociliary protein LOXHD1 as a gene product specifically expressed in EwS. LOXHD1 meets the criteria for a potential oncogene, a diagnostic marker and exquisitely specific tumor antigen for potential adoptive cell-based therapy. Using various high-throughput sequencing and functional studies, we have demonstrated that LOXHD1 is transcribed using an alternative transcription start site (TSS) through EWSR1::FLI1 binding to an upstream *de novo* GGAA microsatellite with enhancer-like properties. Except for its association with DFNB77, a progressive form of autosomal-recessive nonsyndromic hearing loss (ARNSHL) (Grillet et al., 2009), and the requirement of LOXHD1 integrity for auditory hair cell mechanotransduction (Trouillet et al., 2021), very little is known about the role of LOXHD1 in normal cell physiology or cancer due to its undetectable expression in a large majority of normal and cancer cells. To our knowledge, this is the first report studying the function of LOXHD1 outside the inner ear. Our studies implicate LOXHD1 as a major determinant of EwS metastasis through its ability to affect cytoskeletal reorganization, regulate EWSR1::FLI1 function, and hypoxic response through modulation of hypoxia-inducible factor 1α (HIF1α) stability. Together, our work provides strong evidence for LOXHD1 acting as an oncogene and even a potential cell-based immunotherapeutic target in EwS.

## RESULTS

### Discovery of *LOXHD1* as an exquisitely specific EwS target gene

**Figure 1.**
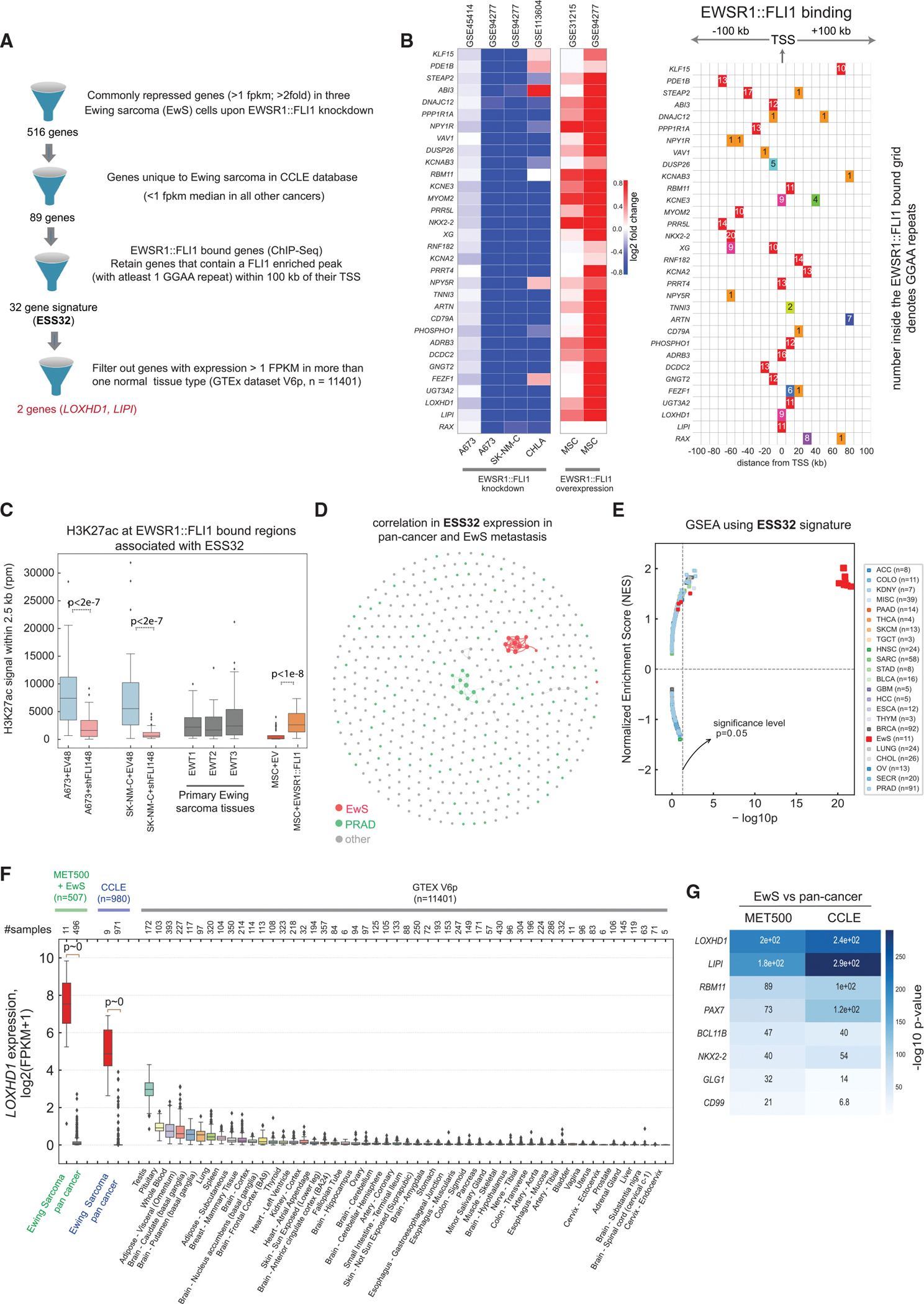
Integrative analysis leading to the identification of ESS32 gene signature, and stereociliary protein LOXHD1 as an EwS-specific gene (A) Flowchart depicting computational pipeline used to identify direct EWSR1:FLI targets in EwS. (B) (Left) log2 fold change in expression (microarray and RNA-seq), for ESS32 in EWSR1::FLI1-knockdown EwS cell lines (n = 4), and EWSR1::FLI1-overexpressing mesenchymal stem cells (MSCs, n = 2). (Right) Table showing the positions of EWSR1::FLI1-bound (ChIP-seq) regulatory regions within ±100 kb of TSS; the numbers denote the number of polymorphic GGAA microsatellite repeats contained within each regulatory element. (C) Increased H3K27ac on ESS32 regulatory region. Total H3K27ac tag density (rpm) within ±2.5 kb of the EWSR1::FLI1-bound regulatory regions for ESS32 is shown in the indicated condition. (D) ESS32 expression identifies EwS among hundreds of pan-cancer metastatic disease. Network plot for the correlation in the expression of ESS32 genes in RNA-seq data of MET500 pan-cancer metastases (n = 500) and metastatic EwS (n = 11). Connectivity is displayed only for samples with Pearson correlation coefficient *r* ≥ 0.5, and the thickness of the connections is proportional to *r*. (E) GSEA analysis of ESS32 in MET500 + EwS RNA-seq data, showing its significant enrichment in >70% of EwS metastatic samples. Abbreviation of sample names can be found in Figure S1D. (F) LOXHD1 is predominantly expressed only in EwS tumors and tumor-derived cell lines. *LOXHD1* expression in MET500 + EwS n = 507, CCLE n = 980, and GTEX n = 11401 transcriptomic datasets. (G) Heatmap shows the log-transformed p values computed for EwS versus pan-cancer samples in MET500 + EwS and CCLE datasets, for the mentioned genes. The p values were computed using an independent, two-sample t test.

To identify highly specific EWSR1::FLI1 target genes with potential oncogenic function, we performed an integrative analysis of various ChIP-seq and transcriptomic datasets and mined for target genes uniquely expressed in EwS. The transcriptomic datasets included cancer cell lines (Cancer Cell Line Encyclopedia, CCLE, n = 980), normal tissues (Genotype-Tissue Expression, GTEx V6p, n = 11401), primary tumors (The Cancer Genome Atlas, TCGA, n = 9205), and pan-cancer metastatic tumor biopsies (MIONCOSEQ Program, MET500 cohort, n = 507) (Robinson et al., 2017). A schematic of this pipeline is shown in Figure 1A, and more information on the datasets used in the analysis can be found in Tables S1 and S2. Using cancer cell line RNA-seq data, we first identified 516 genes that were commonly downregulated (>1 FPKM expression and ≥2-fold down) by EWSR1::FLI1 knockdown in three well-characterized EwS cell lines, SK-N-MC, A673, and CHLA-10 (Gollavilli et al., 2018; Riggi et al., 2014). Similarly, we also identified 176 commonly upregulated genes (>1 FPKM and ≥2-fold up) that could be a representative signature of the EWSR1::FLI1 repressed genes. We next used the CCLE dataset to filter out genes with expression >1 FPKM in any non-EwS cancer cell lines and narrowed our list to 89 highly specific EwS expressed genes (Figure S1A). To qualify these genes as direct EWSR1::FLI1 targets, we used ChIP-seq data for EWSR1::FLI1 enrichment in A673, SK-N-MC, and EWSR1::FLI1-overexpressing mesenchymal stem cells (MSCs), which are believed to be the cell-of-origin for EwS (Figure S1B) (Riggi et al., 2014). Genes that contain at least one EWSR1::FLI1 peak within ±100 kb of their TSS were selected as candidate direct targets. Our analysis identified 32 EwS-specific, EWSR1::FLI1 regulated genes, henceforth called ESS32 (Table S3). Nearly all the genes in this set displayed a pronounced loss of expression upon knockdown of EWSR1::FLI1 in EwS cell lines and a gain of expression in MSCs ectopically overexpressing EWSR1::FLI1 (Figure 1B, left). As an oncogenic pioneer transcription factor, EWSR1::FLI1 binds specifically to GGAA microsatellites repeat sequence and creates *de novo* enhancers from a closed chromatin conformation leading to transcriptional activation of multiple oncogenes (Boulay et al., 2017; Gangwal et al., 2008; Riggi et al., 2014). Interestingly, 75% of the EWSR1::FLI1-bound regions associated with ESS32 contained at least five consecutive GGAA microsatellite repeats (Figures 1B, right, and S1C), indicating strong EWSR1::FLI1 localization at these regions. To determine if these EWSR1:FLI-bound regions for ESS32 are *de novo* enhancers, we analyzed the presence of active enhancer mark, H3K27ac (Figure 1C). H3K27ac was significantly depleted around the ESS32 enhancers in both A673 and SK-N-MC upon EWSR1::FLI1 knockdown and was enriched in MSCs upon overexpression of EWSR1::FLI1; thus, demonstrating that the FLI1-bound enhancers are indeed formed *de novo*. In addition, the three primary EwS tissues showed an enrichment in H3K27ac levels, which are comparable to that in A673 and SK-N-MC cells. To illustrate the EwS-specific nature of ESS32 and its potential as a biomarker for EwS diagnosis, we analyzed the correlation of ESS32 expression among the samples in the MET500 + EwS dataset consisting of pan-cancer metastasis, and 11 EwS metastasis (Figure 1D). The dense connectivity between 10 of 11 EwS samples (Pearson correlation coefficient *r* > 0.5), and the lack of such a strong connectivity with other cancer types supports a high specificity of the ESS32 gene set to EwS. Furthermore, ESS32 gene signature stratified EwS from pan-cancer cohort in a Gene Set Enrichment Analysis (GSEA) (adjusted p value <0.05, normalized enrichment score [NES] >1.5) demonstrating its power and specificity toward EwS, which could be used as diagnostic marker (Figure 1E). The ESS32 gene set is composed of some known EwS genes, such as *KLF15*, *NKX2–2*, *STEAP2*, as well as many new targets that have not yet been associated with EwS (Tables S3 and S4). However, it still presented a weak degree of association with other cancers such as prostate and ovarian cancer. Next, we filtered out genes contributing to this non-specificity using an additional filter based on the TCGA and MET500 + EwS datasets (Table S5), wherein we discarded any genes with >1 FPKM expression in more than one cancer type (Figures S1D and S2A). This narrowed the ESS32 list to three EwS-specific genes, namely *RBM11*, *LIPI*, and *LOXHD1*, of which *RBM11* displayed high expression across multiple tissue types in the GTEx dataset, whereas *LIPI* and *LOXHD1* showed marginal expression only in thyroid and testis, respectively (Figure S2B). Both *LOXHD1* and *LIPI* showed exquisitely restricted expression in the metastatic EwS tissues (Figures 1F and S2C). Remarkably, the specificity of *LOXHD1* and *LIPI* surpassed the commonly used EwS diagnostic markers *CD99*, *NKX2–2*, *PAX-7*, *BCL11B*, and *GLG1* (Baldauf et al., 2018; Orth et al., 2020; Toki et al., 2018; Yoshida et al., 2012) (Figures 1G, S2C, S3A, and S3B). *LIPI* has been reported to be upregulated in EwS, where it is known to regulate lysophosphatidic acid-mediated signaling (Foell et al., 2008; Mahlendorf and Staege, 2013). However, little is known about LOXHD1 expression and function in normal physiology and cancer. To further define the spectrum of LOXHD1 expression in EwS, we queried Affymetrix array data comprising normal, primary, metastasis, and recurrent EwS samples (Postel-Vinay et al., 2012), and found its expression to be significantly upregulated in primary tissue, and trended with disease progression (Figure S3C). To further validate these findings, we profiled 12 primary EwS patient samples for *LOXHD1* using qRT-PCR, of which 10 EwS samples displayed more than a hundred-fold higher *LOXHD1* expression compared with non-EwS samples (Figure S3D). These data strongly suggest *LOXHD1* as a highly specific EwS gene, and led us to investigate the role of LOXHD1 as a mediator driving EwS.

### Mapping short isoform of LOXHD1 transcript and protein in EwS cells

**Figure 2.**
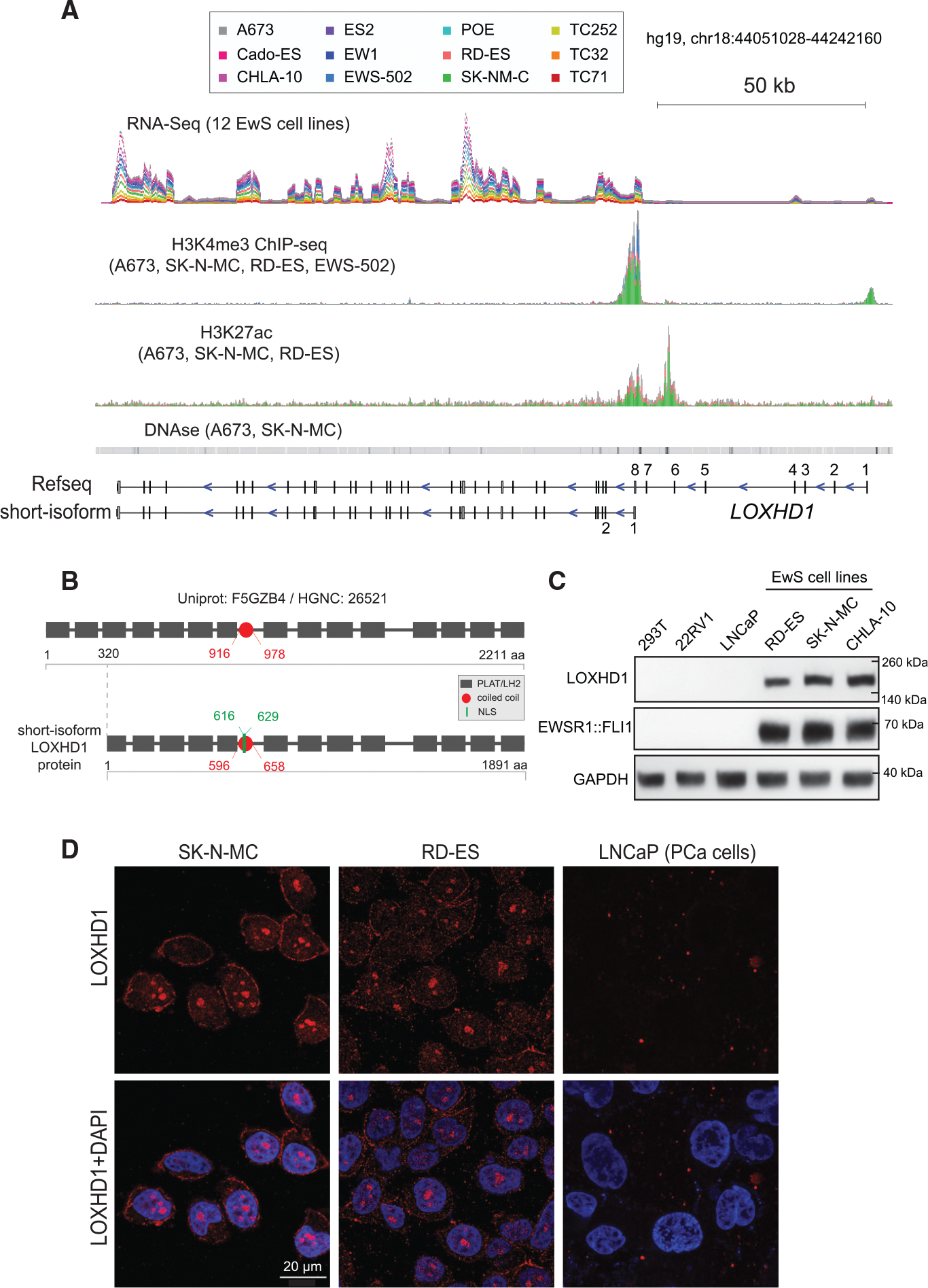
Alternative TSS and identification of NLS in LOXHD1 (A) IGV plot of RNA-seq in EwS cell lines, along with ChIP-seq track for transcription activation mark H3K4me3 and H3K27ac showing alternative TSS for *LOXHD1*. ENCODE DNase 1 hypersensitivity (HS) data for SK-N-MC and A673, showing HS site near the TSS. (B) Protein domain structure for the full-length and short-isoform LOXHD1. Newly identified NLS (nuclear localization signal) and the coiled-coil domain is indicated with aa position. (C) Detection of stereociliary LOXHD1 protein in EwS cells. Immunoblot analysis for LOXHD1 and EWSR1::FLI1 levels in three EwS cells and two prostate cancer cells (LNCaP, 22RV1) and HEK293T cells. GAPDH used as a loading control. (D) Immunofluorescence imaging showing LOXHD1 (red) expression on the plasma membrane and in the nucleus in RD-ES and SK-N-MC cells. DAPI (blue) used to stain the nucleus. LNCaP cells used as a negative control.

*LOXHD1* codes for an evolutionarily conserved protein containing 15 PLAT (polycystin-1, lipoxygenase, alpha-toxin) domains. The biological function of PLAT domains is not well established, but it is speculated that they target proteins to the plasma membrane (Bateman and Sandford, 1999; Grillet et al., 2009). Very little is known about the role of LOXHD1 in normal cell physiology or in cancer, due to its undetectable expression in a large majority of normal or cancer cells. Therefore, we first sought to identify the transcript and protein structure of the LOXHD1 in the EwS cells. The Ensembl GRCh37 annotation of *LOXHD1* gene (ENSG000000167210, chr18: 44056935–44236996) shows 40 exons and multiple splice isoforms. The 6848 -base pair (bp)-long major isoform ENST00000536736 encodes the canonical 2211 amino acids (aa) protein (UniprotKB ID: F5GZB4) with 15 PLAT and one coiled-coil domain. However, our analysis of RNA-seq data from 12 EwS cell lines, with varying levels of expression, showed nearly zero transcriptional output for the first seven exons (Figures 2A and S2A), suggesting that transcription of *LOXHD1* in EwS cells potentially began from an alternative start site skipping the first seven exons. To test this, we integrated ChIP-seq data for H3K4me3 and H3K27ac, with RNA-seq from 12 EwS cell lines to map the EwS-specific *LOXHD1* transcript structure. A common TSS for *LOXHD1* was found in all the 12 EwS cell lines located at the eighth exon in the current Ref. Seq. annotation (Figure 2A). ChIP-seq tracks revealed that the active enhancer/promoter and TSS region for *LOXHD1* locates slightly upstream of exon 8, as evidenced by the pronounced enrichment of both H3K27ac and H3K4me3 mark, respectively in the region around exon 8 (Figure 2A). In addition, the DNase hypersensitivity data for two EwS cell lines A673 and SK-N-MC also showed a DNase 1 hypersensitive region near our newly annotated H3K4me3-bound TSS, and H3K27ac-bound regulatory elements (Figure 2A). Furthermore, we carried out 5′ rapid amplification of cDNA ends (RACE) assay in EWSR1::FLI1-positive SK-N-MC, RD-ES, and EWSR1::ERG-positive CADO-ES1 cells and confirmed the LOXHD1 short-isoform TSS at exon 8 (Figure S2C). The short isoform LOXHD1 contains 33 exons and codes for 1891 aa protein containing 13 PLAT and 1 coiled-coil domains (Figure 2B). We predicted the coiled-coil structure for amino acids 596 through 658 in the remapped protein using the COILS server (Lupas et al., 1991) (Figures 2B and S2C) in accordance with a previous prediction from mouse cDNAs (Grillet et al., 2009). In addition, using the NLS (nuclear localization signal) mapper (Kosugi et al., 2009), we identified a cryptic NLS (aa 616–629) within the coiled-coil region (Figure S2D). Immunoblot analysis in a panel of EwS (n = 3) and non-EwS (n = 3) cell lines using LOXHD1-specific antibody (Grillet et al., 2009) displayed a specific band between 200 and 220 kDa in EwS cells (Figure 2C), which is consistent with the predicted molecular weight of a 1891 aa (216.4 kDa) short-isoform LOXHD1 protein. The presence of coiled-coil and NLS domain suggested nuclear localization of LOXHD1 in addition to its cytosolic functions. To test whether LOXHD1 localizes to the nucleus, we cloned a portion of mouse *Loxhd1* exon19 which is homologous to human *LOXHD1* with over 92% sequence similarity and encompasses both the NLS and coiled-coil domains (Figure S2E). Overexpression of the HA-tagged LOXHD1 exon19 in 293T cells showed strong immunofluorescence staining in the cell nucleus (Figure S2E). We further confirmed this feature through immunofluorescence staining of the endogenous LOXHD1 in the EwS cell lines. In SK-N-MC and RD-ES cells, LOXHD1 staining was observed on both the plasma membrane/cytoplasm and in the nucleus, whereas prostate cancer cell line LNCaP used as a negative control displayed no specific staining (Figure 2D). Together, these data demonstrate an alternative TSS for *LOXHD1* in EwS cells and provide evidence for its protein expression in both cytoplasmic and nuclear compartments.

### EWSR1::FLI1 binding to the GGAA microsatellite creates *de novo* enhancer upstream of LOXHD1 and regulates its expression

**Figure 3.**
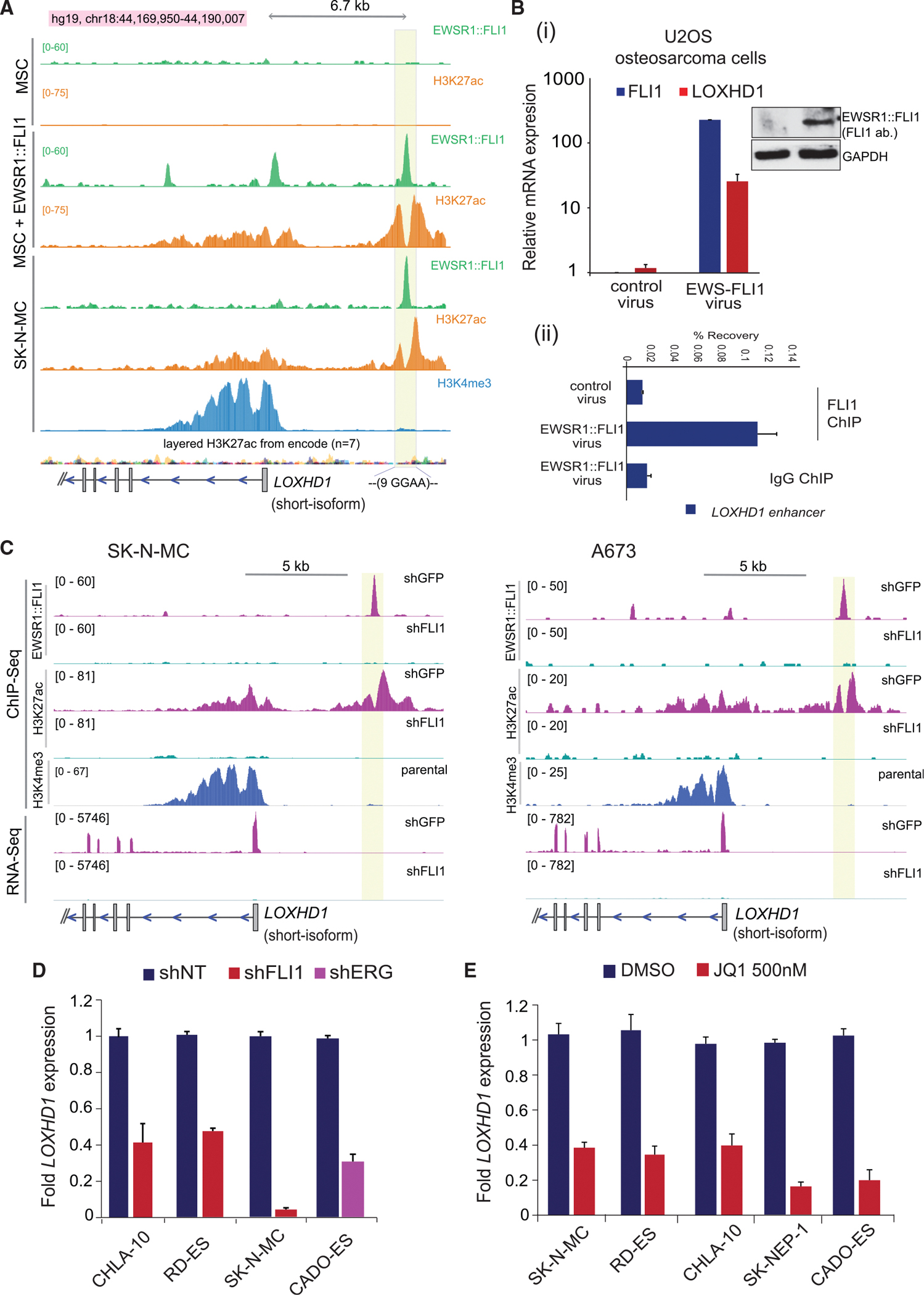
EWSR1::FLI1 binding to the polymorphic GGAA microsatellite creates a *de novo* enhancer upstream of *LOXHD1* and regulates its expression (A) Genome browser ChIP-seq tracks showing the EWSR1::FLI1 binding to *LOXHD1* upstream region containing GGAA repeats, generating a *de novo* enhancer marked by H3K27ac in EWSR1::FLI1 overexpressing MSC, and wild-type SK-N-MC cells (Gene Expression Omnibus [GEO]: GSE94278). (B) (i) Ectopic expression of EWSR1::FLI1 leads to transcriptional activation of LOXHD1 in non-EwS cancer cells. qRT-PCR showing upregulation of LOXHD1 in U2OS osteosarcoma cells upon EWSR1::FLI1 expression, which was detected using FLI1 primers, n = 3. Immunoblot for EWSR1::FLI1, and GAPDH (loading control) in the indicated samples. (ii) ChIP-qPCR analysis showing direct binding of EWSR1::FLI1 on the enhancer site of *LOXHD1* in U2OS cells. n = 3. (C) Knockdown of EWSR1::FLI1 collapses the *de novo* enhancer leading to silencing of *LOXHD1* expression. Genome browser view of integrated ChIP-seq and RNA-seq tracks showing loss of EWSR1::FLI1 enrichment to the GGAA microsatellite with a concomitant loss of H3K27ac and transcriptional silencing of LOXHD1, respectively, upon shFLI1-mediated EWSR1::FLI1 knockdown in SK-N-MC (left) and A673 cells (right). ChIP-seq track for H3K4me3 denotes the TSS (GEO: GSE94278). (D and E) EWSR1::ETS fusion knockdown or inhibition of its co-activator BRD4 downregulates *LOXHD1* expression. qRT-PCR showing downregulation of *LOXHD1* transcript upon short hairpin RNA (shRNA)-mediated EWSR1::FLI1/EWSR1::ERG knockdown or treatment with 500 nM JQ1 for 24 h in a panel of EwS cell lines, n = 3 technical replicates.

As a pioneer transcription factor, EWSR1:: FLI1 binds specifically to GGAA microsatellite repeat sequences and creates *de novo* enhancers leading to transcriptional activation of multiple oncogenes (Boulay et al., 2017; Gangwal et al., 2008; Riggi et al., 2014). The potential regulatory region for *LOXHD1* is located roughly 6.7 kb upstream of the newly annotated TSS and contains nine consecutive GGAA repeats (Figure 1B). To demonstrate the presence and *de novo* nature of enhancer regulating *LOXHD1* expression, we analyzed ChIP-seq data for MSCs in control and EWSR1::FLI1 overexpression conditions (Riggi et al., 2014) (Figure 3A). Ectopic expression of EWSR1::FLI1 displayed *de novo* enhancer formation characterized by H3K27ac deposition and a strong EWSR1::FLI1 peak at ~6.7 kb upstream of *LOXHD1* TSS; and transcription of *LOXHD1* (Figures 3A and S3A). As expected, the same site was occupied by endogenous EWSR1::FLI1 with enriched H3K27ac in SK-N-MC cells (Figure 3A). Similar to this, a *de novo* enhancer spaced between *LIPI* and *RBM11* was observed, which might regulate their expression (Figure S3B). These observations indicate that LOXHD1, which is transcriptionally silent in a vast majority of normal and pan-cancer cells, is induced exclusively in EwS by the pathognomonic oncofusion EWSR1::ETS including EWSR1::FLI1. Consistent with these findings, ectopic expression of EWSR1:FLI in U2OS osteosarcoma cells led to its binding to *LOXHD1* enhancer resulting in *LOXHD1* expression (Figure 3B). Likewise, in an orthogonal approach, EWSR1::FLI1 knockdown in SK-N-MC and A673 cells resulted in the disassembly of the *LOXHD1* enhancer with a complete loss of H3K27ac and a resulting loss in the expression of *LOXHD1* (Figure 3C). As expected, BAF155, an SWI/SNF complex component known to get recruited to EWSR1::FLI1-bound enhancers (Boulay et al., 2017), was found to be enriched at the GGAA site upstream of *LOXHD1* upon overexpression of EWSR1::FLI1 in MSC or lost upon its knockdown in SK-N-MC cells (Figure S3C). To further validate the role of EWSR1::ETS in regulating *LOXHD1* expression, we knocked down EWSR1::FLI1 or EWSR1::ERG in a panel of EwS cells and observed 2- to 10-fold decrease in *LOXHD1* expression (Figures 3D and S3D). In addition, inhibiting BET bromodomain, which we previously demonstrated to be important for EWSR1::ETS-mediated transcription (Gollavilli et al., 2018), with JQ1 in EwS cells resulted in the loss of *LOXHD1* expression (Figure 3E), further indicating the role of EWSR1::ETS and its associated transcriptional complex in LOXHD1 transcription. These observations demonstrate transcriptional regulation of *LOXHD1* through a distal *de novo* enhancer assembled by the pathognomonic EWSR1::ETS transcription factor in EwS.

### Genomic deletion or epigenetic silencing of the EWSR1::FLI1 bound *de novo* enhancer represses *LOXHD1* transcription

**Figure 4.**
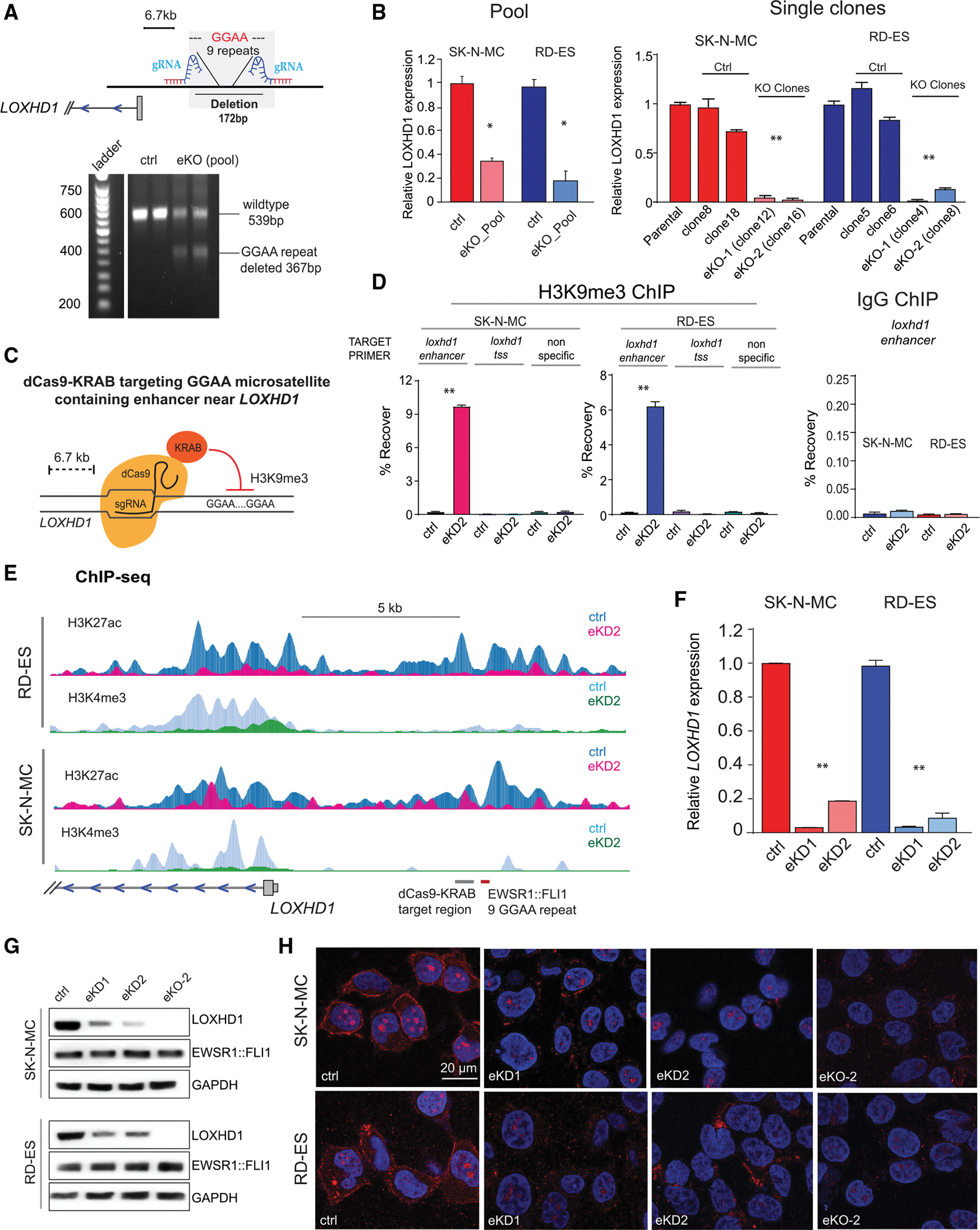
Knockout or dCas9-KRAB-mediated silencing of EWSR1::FLI1-bound *de novo* enhancer quashes LOXHD1 transcription (A) (Above) Schematic showing the CRISPR sgRNA specifically flanking the GGAA microsatellite upstream of LOXHD1. (Below) DNA ethidium bromide-stained gel image showing the deletion of GGAA repeats in cells transduced with enhancer targeting sgRNA or Cas9 control lentivirus. (B) qRT-PCR showing *LOXHD1* expression in enhancer knockout pools (left) and two independent isogenic single-cell clones (right) compared with their respective parental controls and single-cell clone controls. n = 3. (C) Schematic showing the CRISPR-dCas9-KRAB (CRISPRi)-mediated epigenetic silencing of GGAA microsatellite containing *LOXHD1* enhancer. Two independent small guide RNAs (sgRNA1 and sgRNA2) were designed adjacent to the GGAA microsatellite. (D) ChIP-qPCR analysis showing accumulation of H3K9me3 at the *LOXHD1* upstream enhancer (m.s. region primers) in cells expressing dCas9-KRAB and sgRNA2 (eKD2, enhancer KnockDown 2). LOXHD1 TSS primers and nonspecific (n.s.) region primers were used as negative control and immunoglobulin (Ig) G served as ChIP negative control. n = 3. (E) Depletion of active transcription marks upon *de novo* enhancer knockdown. ChIP-seq tracks of H3K27ac and H3K4me3 signals at the *LOXHD1* loci in cells expressing dCas9-KRAB and sgRNA2 as in (D). gRNA target site and EWSR1::FLI1 target microsatellite region is indicated with thick gray and red line, respectively. (F) qRT-PCR showing the loss of *LOXHD1* expression in enhancer knockdown cells. n = 3 technical replicates. (G) Immunoblots showing loss of LOXHD1 protein in enhancer knockdown (dCas9-KRAB) or enhancer knockout (CRIPSR-cas9) cells. EWSR1::FLI1 and GADPH were used as control. (H) Immunofluorescent staining of LOXHD1 (red). The nucleus was visualized by DAPI (blue). *p < 0.05, **p < 0.001 by two-tailed Student’s t test.

We next investigated the functional association between *LOXHD1* expression and its EWSR1::FLI1-bound upstream enhancer by deleting the GGAA microsatellite repeats through CRISPR-Cas9 genome editing. Infection with single guide RNA (sgRNA) Cas9 lentivirus targeting regions on either side of the GGAA repeat led to the deletion of approximately 172 bp DNA (Figures 4A and S4A) and a concomitant decrease in the transcription of *LOXHD1* in a pool population of SK-N-MC and RD-ES cells (Figure 4B). In addition, we observed >90% reduction in *LOXHD1* mRNA levels in independent single clones containing the heterozygous enhancer deletion (eKO1 and eKO2) (Figures 4B right, S4B, and S4C). In an orthogonal set of experiments, we silenced the activity of the *LOXHD1* enhancer using the CRISPR dCas9-KRAB system (CRISPRi) with two independent sgRNAs (eKD1 and eKD2) targeting the adjacent region of the EWSR1::FLI1 bound GGAA repeat (Figure 4C). CRISPRi induces focal chromatin-repressive states by KRAB-mediated H3K9me3 deposition at target sites (Gilbert et al., 2014; Thakore et al., 2015). We first assessed and found the accumulation of H3K9me3 within the targeted region in cells transduced with CRISPR dCas9-KRAB sgRNA constructs (Figure 4D). Consistent with histone deacetylase activity of the KRAB domain, ChIP-seq analysis of H3K27ac demonstrated the loss of signal from the targeted microsatellite and adjacent region (Figure 4E). In addition, reduction in H3K4me3 and H3K27ac from the *LOXHD1* TSS was observed in CRISPRi cells when compared to controls, suggesting that the chromatin state changes on the distal enhancer could affect the active transcription mark likely due to the loss of enhancer-promoter contact (Figure 4E). As expected, compared to control cells, repression of the enhancer led to significant loss of *LOXHD1* transcription (Figure 4F). These results were further confirmed by immunoblotting and immunofluorescence analyses that demonstrated the reduction of LOXHD1 protein in the eKD polyclonal pools and eKO single-cell clones compared with controls (Figures 4G and 4H). Altogether, our findings from genetic deletion and epigenetic silencing approaches provide substantial evidence that *LOXHD1* expression is regulated by a distal EWSR1::FLI1-bound *de novo* enhancer in EwS.

### LOXHD1 silencing impairs major oncogenic transcription factor response and cytoskeletal organization

**Figure 5.**
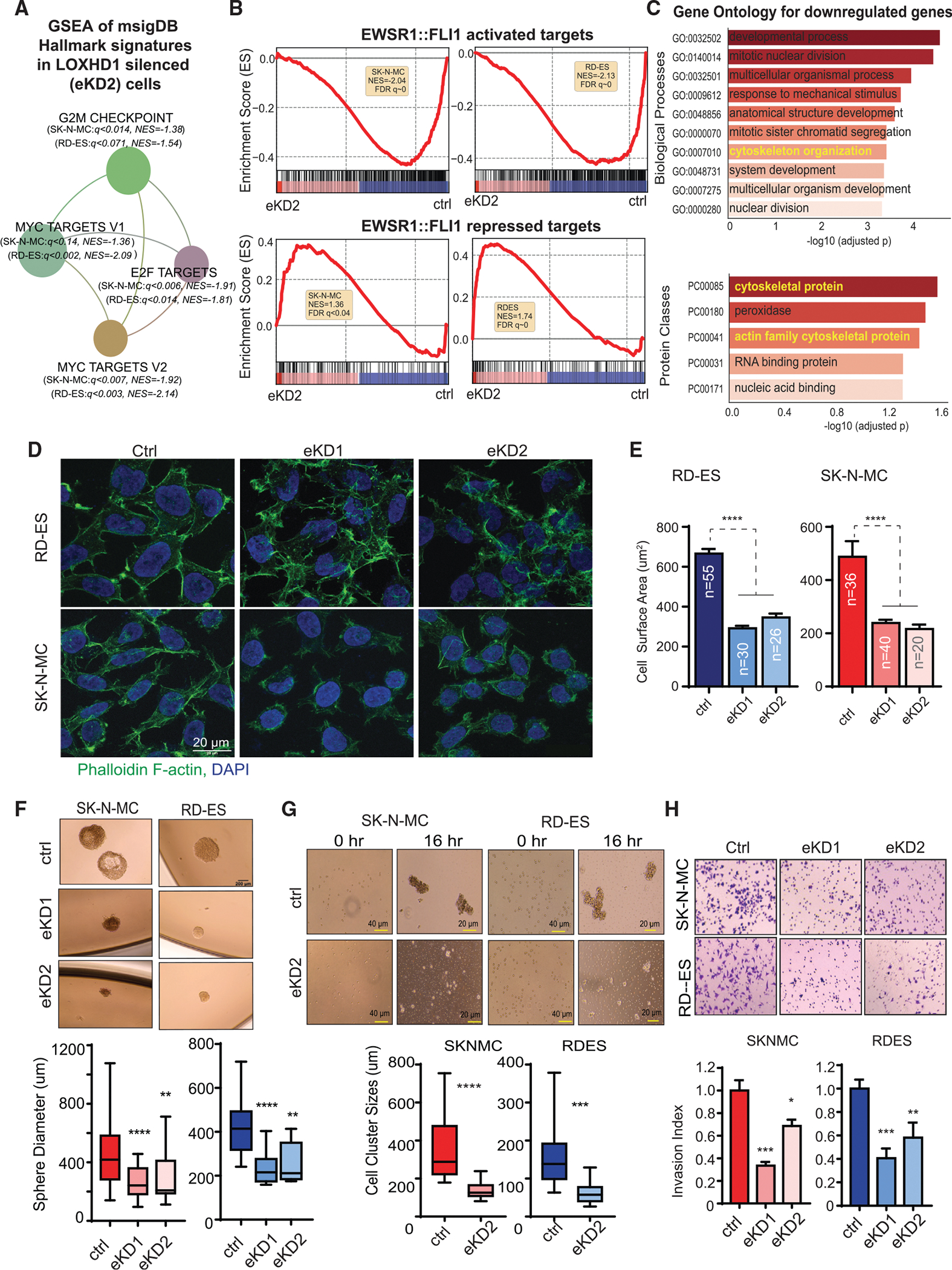
LOXHD1 loss impairs major oncogenic transcription factor response and cytoskeletal organization (A) RNA-seq followed by GSEA analysis of hallmark signatures show four common negatively enriched pathways in LOXHD1-silenced RD-ES and SK-NM-C cells. The individual values of FDR q and NES are shown. (B) GSEA plots showing negative enrichment of the 516 EWSR1::FLI1-activated genes and positive enrichment of the 176 EWSR1::FLI1-repressed genes in the *LOXHD1* eKD2 cells. (C) Gene Ontology (GO) terms of biological process and protein classes for common downregulated genes in *LOXHD1* eKD2 cells. (D and E) Cytoskeletal disorganization in LOXHD1 silenced cells. (D) Representative images of Phalloidin F-actin and DAPI staining in cells with and without LOXHD1 silencing. (E) Quantification of cell surface areas in the immunofluorescence staining images with ImageJ. (F) LOXHD1-silenced cells display reduced anchorage-independent growth. Representative images and quantification of a sphere formation assay performed with indicated cells embedded in 50% of Matrigel in a 24-well plate. SK-N-MC ctrl n = 75; eKD1 n = 15; eKD2 n = 14. RD-ES ctrl n = 20; eKD1 n = 10; eKD2 n = 5. (G) LOXHD1 silenced cells display reduced cell aggregation property. Representative images and quantification of a cell aggregation assay performed by seeding single-cell suspension on poly-HEMA-coated ultralow attachment plates. Images were taken at 0 h and 16 h. SK-N-MC ctrl n = 21; eKD2 n = 28. RD-ES ctrl n = 46; eKD2 n = 40. (H) LOXHD1-silenced cells display reduced Matrigel invasion. Representative images (n = 3) and quantification of invaded cells 48 h post plating are shown for control and *LOXHD1* enhancer knockdown cells. ****p < 0.0001, ***p < 0.001, **p < 0.01, *p < 0.05 by two-tailed Student’s t test.

Except for a study showing that a missense mutation in mouse *LOXHD1* affects the function of the sensory cells involved in hearing (Grillet et al., 2009), not much is known about its role in normal or cancer cell physiology. Toward this end, we first attempted to understand the consequence of LOXHD1 loss in the EwS cells by performing RNA-seq in parental versus enhancer knockdown (eKD) cells. The eKD cells displayed significant changes in the transcriptome with hundreds of genes up- and downregulated as a result of LOXHD1 silencing (Figure S5A). GSEA analysis identified common negative enrichment of oncogenic hallmark MYC signatures, G2M checkpoint, and E2F targets in LOXHD1 silenced cells (Figure 5A). Furthermore, the EWSR1::FLI1-target genes (Figure 1A) and the ESS32 gene set were both negatively enriched and EWSR1::FLI1-repressed genes (identified as genes that show at least 2-fold upregulation upon EWSR1::FLI1 knockdown in the data shown in Figure 1A) show positive enrichment in these cells (Figures 5B and S5B). Together these data suggest that LOXHD1 silencing may have a negative effect on the driver transcriptional programs and tumorigenic potential of EwS cells. In addition, Gene Ontology (GO) pathway enrichment analysis showed cytoskeleton organization and actin family protein among the top deregulated biological pathways (Figure 5C). Plasma membrane-associated proteins regulate cytoskeletal assembly through their ability to regulate components of the actin and microtubule filament network (Fletcher and Mullins, 2010). Reorganization of cytoskeleton affects cell signaling, polarity, motility, cell-cell, and cell-ECM (extracellular matrix) interactions and, more importantly, alters the metastatic potential of cancer cells (Khanna et al., 2004; Luna and Hitt, 1992). Based on the above observations and given the fact that LOXHD1 primarily was found to be associated with the plasma membrane (Grillet et al., 2009), we hypothesized that LOXHD1 regulates EwS cell cytoskeleton and promotes tumorigenesis. Immunofluorescent staining of F-actin displayed relatively organized cytoskeletal structure underneath the plasma membrane with elongated nuclear morphologies representative of spread, adherent cells (Figure 5D). However, LOXHD1-silenced eKD1 and eKD2 cells displayed diffuse, highly irregular cytoskeletal patterns with circular nuclear morphologies representative of non-adherent cells. The cell surface area, which is directly related to the degree of cellular adhesion to its substrate, for eKD1 and eKD2 cells, was substantially smaller compared with its controls (Figure 5E). The data indicated that the silencing of LOXHD1 alters the cell-to-cell and cell-to-matrix interactions. We then hypothesized that cell growth at single-cell density, which requires optimum cell-to-cell contact, could be compromised in these cells, and tested it by sphere formation on 3D Matrigel, and 2D colony formation assays. As expected, LOXHD1-silenced cells form substantially less and smaller spheres/colonies than parental controls (Figures 5F and S5C). We further tested the ability of eKD cells to form aggregates by suspending them in ultralow attachment plates. While the parental cells exhibited stabilized, large aggregates containing hundreds of cells, the eKD cells did not display similar aggregates even 16 h post plating, suggesting a reduced cell-cell contact potential as a result of altered cytoskeleton in the LOXHD1-silenced cells (Figure 5G). Since the cytoskeletal organization can significantly alter the migratory potential of cancer cells, we performed wound healing and Boyden chamber invasion assays. We found significantly reduced migration and invasion of LOXHD1 silenced cells compared with the respective parental controls (Figures 5H, S5D, and S5E). Notably, we did not observe any change in the proliferation of LOXHD1-silenced cells in 2D cell culture in 48- and 96-h time, though a slightly reduced growth was observed upon 6-day time point (Figure S5F). This was further associated with increased G1 phase and reduced S and G2-M phase cells (Figure S5G). Together, these data demonstrate that LOXHD1 silencing in EwS cells impairs major oncogenic transcription factor pathways, including EWSR1::FLI1, cytoskeletal organization, and cell cycle progression, leading to reduced anchorage-independent growth and metastatic potentials *in vitro*.

### LOXHD1 knockdown attenuates hypoxia response in EwS cells by destabilizing HIF1α

**Figure 6.**
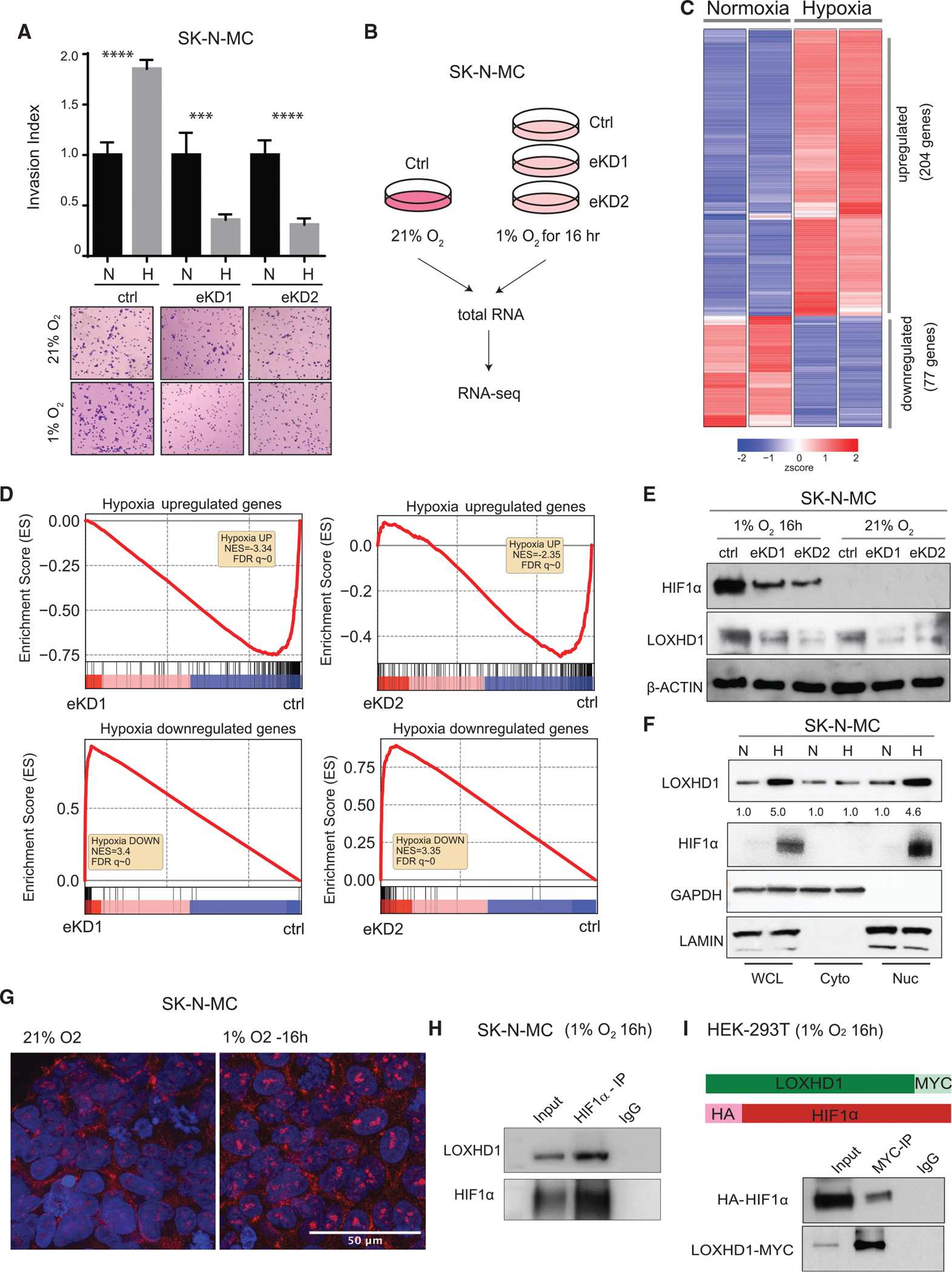
LOXHD1 silencing impairs EwS response to hypoxia (A) Hypoxia induces impaired invasion response in LOXHD1 silenced cells. n = 3. (Top) Quantification of Matrigel invasion assay performed with control, eKD1, and eKD2 cells at 21% O_2_ and 1% O_2_ for 24 h. (Bottom) Representative images of each indicated group. (Quantification of the hypoxia-only condition is shown in Figure S8B). (B) Schematic showing the hypoxia RNA-seq experimental design. (C) Hypoxia induces major transcriptional changes in LOXHD1 intact EwS cells. Heatmap shows differential expressed genes under hypoxia (1% O_2_ for 16 h) compared with normoxia in SK-N-MC cells. n = 2 biological replicates. (D) LOXHD1 silencing reverses hypoxia response. GSEA showing negative enrichment of the hypoxia upregulated signature and positive enrichments of the hypoxia downregulated signature in *LOXHD1* eKD1 and eKD2 cells. (E) LOXHD1 silencing reduces HIF1α stabilization under hypoxia. Immunoblots of HIF1α and LOXHD1 in control and eKD cells under hypoxia and normoxia culture. β-actin used as loading control. (F) Hypoxia promotes nuclear LOXHD1 enrichment. Immunoblots for LOXHD1 and HIF1α in SK-N-MC whole cell (WCL), cytoplasmic (Cyto), and nuclear (Nuc) lysates after 16 h of normoxia (N) and hypoxia (H) culture. GAPDH and LAMIN were used as markers for cytoplasmic and nuclear compartment, respectively. Quantification of LOXHD1 levels is shown. (G) Immunofluorescent staining of LOXHD1 showing enriched nuclear expression under hypoxia. (H) LOXHD1 interacts with endogenous HIF1α in SK-N-MC cells. Cells were incubated at 1% O_2_ for 16 h, nuclear lysate was used for immunoprecipitation with HIF1α antibody or IgG control. (I) Interaction between LOXHD1 and HIF1α HEK293T cells. Cells were co-transfected with MYC-tagged LOXHD1 and HA-tagged HIF1α and cultured under hypoxia for 16 h. Total protein lysates used for immunoprecipitation with MYC-tag antibody. (Top) Schematic showing the structure of the two constructs. (Bottom) Immunoblots of anti-HA and anti-Myc antibodies.

Intratumoral hypoxia is a common feature of solid malignancies including sarcomas. Hypoxia-inducible factor (HIF) proteins, mainly HIF1α and HIF2α, are transcription factors essential for cellular adaptation to hypoxic stresses (Majmundar et al., 2010). Overexpression of HIF1α has been shown to enhance the metastatic potential of sarcomas, including EwS (Brizel et al., 1996; Eisinger-Mathason et al., 2013; El-Naggar et al., 2015). Remarkably, the invasive potential of LOXHD1-proficient EwS cells was amplified under hypoxic conditions confirming earlier reports that hypoxia promotes sarcoma invasion and metastasis (Aryee et al., 2010; Brizel et al., 1996; Eisinger-Mathason et al., 2013; El-Naggar et al., 2015) (Figures 6A, S6A, and S6B). In contrary, LOXHD1-silenced cells displayed a reduced invasion capacity in hypoxic culture conditions without affecting proliferation (Figures 6A and S6A). The difference in the invasion capacity between control and knockdown cells was far more dramatic in the hypoxic condition than in the normoxia culture (Figures 5H and S6B). These observations indicated that LOXHD1 may play a role in EwS response to hypoxia. Since LOXHD1 protein was found in the nuclear compartment of the EwS cells, we wondered whether it has a role in HIF1α transcriptional output. Therefore, to better understand this process, we studied hypoxia-induced transcriptome changes in parental and LOXHD1-silenced SK-N-MC cells using RNA-seq (Figure 6B). Using differential expression analysis (Love et al., 2014) for the hypoxic samples, we first identified 204 genes with >4-fold upregulation and 77 genes with >4-fold downregulation (adjusted p value <0.001), suggesting a robust transcriptional response to hypoxia in the parental SK-N-MC cells (Figure 6C). As expected, GSEA showed strong positive enrichment for the hallmark hypoxia signature (Figure S6C). The GO for the 204 upregulated genes comprised processes and pathways associated with hypoxic response and HIF-1 signaling (Figure S6D), confirming that the SK-N-MC cells are sensitive to hypoxia. We next evaluated the role of LOXHD1 in EwS hypoxic response in the eKD1 and eKD2 cells. Unlike the controls, the two independent LOXHD1-silenced polyclonal pools showed weaker hypoxic stress response (Figure S6E). GSEA analysis showed a significant reversal of both the upregulated and downregulated gene signature (Figure 6D), suggesting an attenuated hypoxia response in LOXHD1-silenced EwS cells.

HIF1α is the main transcription factor involved in the transcriptional response to hypoxia (Wang et al., 1995). Under hypoxic conditions, HIF1α is primarily stabilized by functional inactivation of prolyl hydroxylases (PHDs) and VHL E3 ligase complex, which label HIF1α for proteasomal degradation (Maxwell et al., 1999). Remarkably, hypoxia-treated eKD1 and eKD2 cells showed less HIF1α protein than controls (Figure 6E) despite a lack of downregulation of its mRNA expression (Figure S6F). In fact, there was a slight increase in *HIF1A* mRNA expression in the hypoxia-treated eKD cells, which could potentially be a result of compensation to restore HIF1α protein in these cells. These data suggest that LOXHD1 silencing most likely destabilizes nuclear HIF1α protein rather than reduces the transcription of *HIF1A*. To test this further, we first investigated the subcellular localization of LOXHD1 by performing cellular fractionation and examined its levels with or without hypoxia. As expected, the LOXHD1 protein was detected in both cytoplasmic and nuclear fractions (Figure 6F), consistent with data shown in Figures 2D and 4H. Interestingly, the nuclear LOXHD1 was enriched in response to hypoxia, which was also apparent in immunofluorescence staining of the endogenous LOXHD1 and colocalized with HIF1α (Figures 6G and S6G). Further, to determine if LOXHD1 mediates HIF1α stabilization, we treated the control and eKD cells with an iron chelator desferrioxamine (DFO), which disrupts the function of iron-dependent PHDs in maintaining HIF1α stabilization (Epstein et al., 2001; Semenza, 2012) and found no differential stability of HIF1α in eKD cells compared with controls (Figure S6H). In addition, inhibiting proteasomal-mediated HIF1α degradation by MG132 rescued the lower HIF1α protein levels seen in eKD cells under hypoxia (Figure S6I). These data suggest a potential deficiency of non-canonical HIF1α regulatory signaling in the LOXHD1-silenced cells. To test whether LOXHD1 interacts with HIF1α protein, we carried out co-immunoprecipitation experiments with nuclear extracts from SK-N-MC cell culture under hypoxia and found the interaction between the endogenous HIF1α and LOXHD1 (Figure 6H). In addition, co-transfection of HA-tagged HIF1α and Myc-tagged LOXHD1 in 293T fibroblasts grown under hypoxic conditions, followed by immunoprecipitation with MYC-tag LOXHD1, was able to pulldown HA-tagged HIF1α (Figure 6I), suggesting a direct physical interaction between these two proteins. Together, these results demonstrate that LOXHD1 functions as a regulator of HIF1α stability and activity in EwS cells.

### LOXHD1 silencing affects EwS metastasis and tumor growth *in vivo*

**Figure 7.**
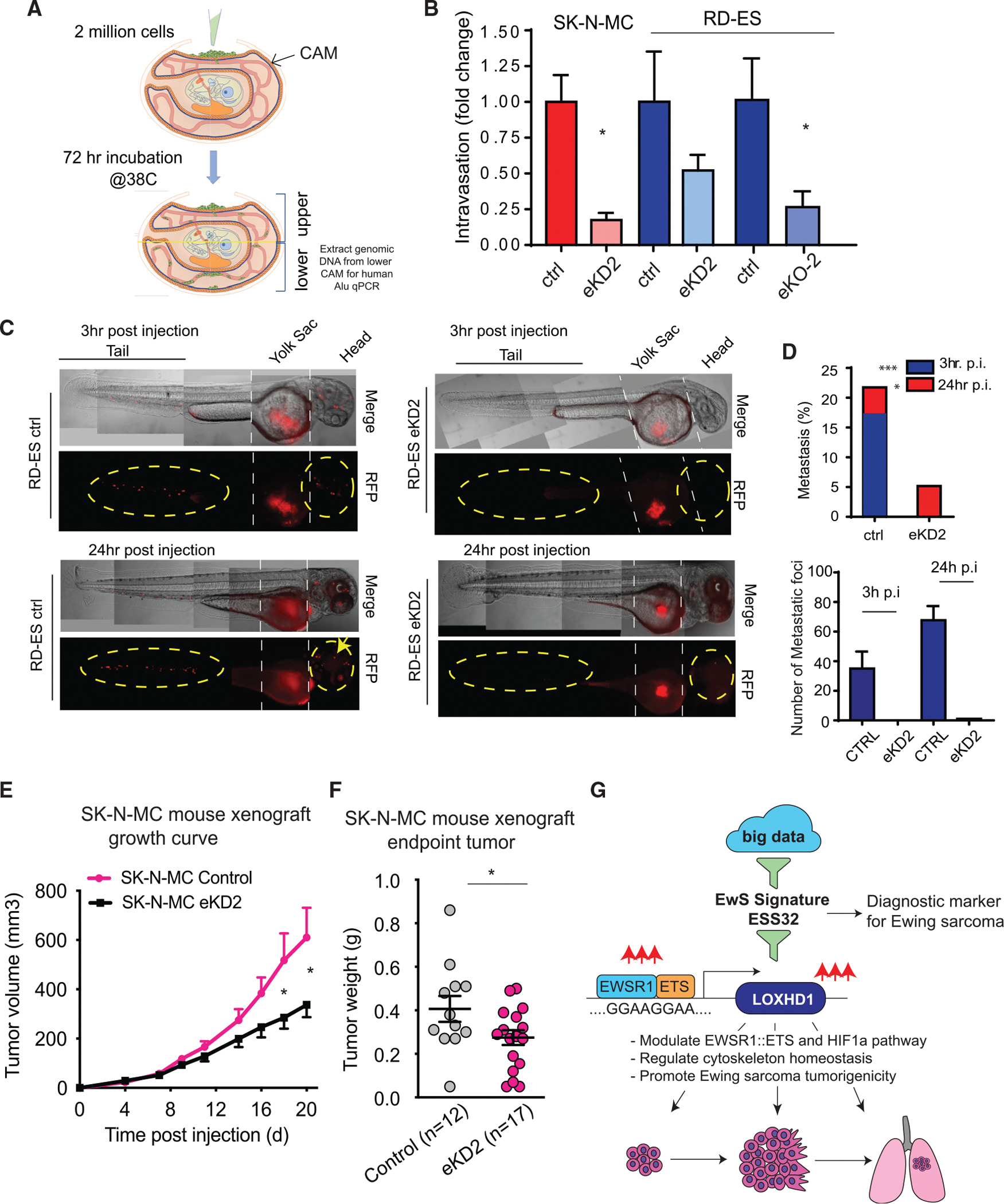
LOXHD1 silencing attenuates the oncogenic and metastatic phenotype of EwS cells *in vivo* (A) LOXHD1 silencing reduces cell intravasation in a chicken CAM model. Schematic showing CAM intravasation assay. Two million cells are cultured atop the embryonic chick upper CAM for 3 days followed by genomic DNA isolation from the lower CAM, which is used to measure the intravasated human cells by qPCR using human-specific Alu primers. (B) Bar graph showing normalized fold difference in the intravasated cells for the indicated group. n = 8 for each group, *p < 0.01, by Student’s t test. (C) LOXHD1 silencing impairs EwS metastasis in a zebrafish model. Representative images of zebrafish in the control and LOXHD1 silenced group showing metastasis in yellow circles and arrow at 3 h and 24 h post-injection. ctrl n = 46; eKD2 n = 58. (D) (Top) Bar graph of percentages of zebrafish harboring metastasis at 3-h and 24-h time point. (Bottom) Quantification of total number of metastatic foci. ***p < 0.001, *p < 0.01 by chi-square test. (E) LOXHD1 silencing attenuates tumor formation in mice. Growth curve of the xenograft experiment using SK-N-MC control and isogenic eKD2 cells. ctrl n = 12; eKD2 n = 17. (F) Bar graph of the endpoint tumor weights. (G) Schematic illustrating the discovery and the role of LOXHD1 in influencing multiple oncogenic pathways in Ewing sarcoma genesis and progression. **p < 0.01, by Student’s t test. n = biological replicates.

Finally, to examine the role of LOXHD1 in EwS tumor growth and metastasis *in vivo*, we used three different metazoan models, namely the chicken chorioallantoic membrane (CAM) model, zebrafish model, and mouse xenograft model. In the CAM assay, cancer cells introduced on the upper CAM proliferate, invade the basement membrane, intravasate the nearby vasculature, and circulate in the blood vessels that can be captured at the lower CAM, thereby providing an estimate of their invasion and intravasation potential (Figure 7A) (Asangani et al., 2013; Lokman et al., 2012; Subauste et al., 2009). We observed a significantly impaired invasion and intravasation to the lower CAM by LOXHD1-silenced cells than their respective parental controls (Figure 7B). We next studied EwS metastasis in a zebrafish model, which has been widely used to test the metastatic potential of various human cancer cell lines, including EwS (El-Naggar et al., 2015; Teng et al., 2013). Strikingly, the zebrafish embryos displayed a significantly impaired metastatic dissemination of RFP-labeled LOXHD1-silenced cells from the yolk sac to the tails and head of the embryos, than the parental controls, providing strong evidence for LOXHD1 as a mediator of EwS metastasis *in vivo* (Figures 7C and 7D). Thereafter, we tested the effect of LOXHD1 silencing in the murine SK-N-MC xenograft model. Compared with parental control, the LOXHD1-silenced SK-N-MC xenograft demonstrated a significantly reduced tumor growth (Figures 7E and 7F), which was accompanied by increased necrotic margins and lower mitotic foci (Figures S7A and S7B). Therefore, the *in vivo* xenograft result is in agreement with the cell cycle progression, colony, and sphere formation assay performed *in vitro*, and together provides concrete evidence supporting that LOXHD1 promotes EwS tumorigenicity. Together, these *in vivo* data clearly establish the role of LOXHD1 in regulating EwS tumor formation and metastasis (Figure 7G).

## DISCUSSION

In this study, we have identified the stereociliary protein LOXHD1 as a highly specific EwS gene product with oncogenic and metastasis-promoting properties. Our results demonstrate the EWSR1::FLI1-mediated *de novo* enhancer activates the expression of this developmentally silenced gene. While previous work has established LOXHD1 mutation in DFNB77, a progressive form of ARNSHL, and also induced mechanotransduction defect in cochlear hair cells (Grillet et al., 2009; Trouillet et al., 2021), we provide the first evidence of its role in cellular physiology and, in particular, EwS tumorigenicity. In addition, through integrative transcriptomic analysis, the identification of ESS32 gene signature comprising known and new EWSR1::FLI1 targets that accurately stratify metastatic EwS from non-EwS samples is a critical discovery with translational potential as an EwS diagnostic and prognostic biomarker.

Besides LOXHD1, there are close to 20 genes in the human genome that code for proteins containing the PLAT domain, including PKD1, and RAB6IP1. With the exception of LOXHD1 that contains multiple PLAT domains, all other proteins possess a single PLAT domain. The highly conserved PLAT domain is involved in protein-protein and protein-lipid interactions (Xu et al., 2016). Usually, they tend to associate peripherally with the cytosolic side of the plasma membrane and mediate interactions with other transmembrane signaling proteins (Bateman and Sandford, 1999). Interestingly, LOXHD1 is required for hearing in humans and mice, and localizes between the membrane and the actin-cytoskeleton of stereocilia, potentially to connect them (Grillet et al., 2009; Trouillet et al., 2021). Although EWSR1::FLI1 was reported to promote cytoskeletal disorganization through regulating zyxin and integrins coding genes (Chaturvedi et al., 2014), the disorganization observed in LOXHD1-silenced cells could be due to (1) potential direct interaction and stabilization of cytoskeletal protein by LOXHD1, or (2) effect of LOXHD1 on EWSR1::FLI1 transcription activity regulating the expression of cytoskeletal genes. Further work is needed to dissect the exact contribution of the multiple PLAT domains of LOXHD1 in protein-protein and lipid-protein signaling, but our work highlights its potential role in cytoskeleton organization. In addition to the 13 PLAT domains, we found a coiled-coil domain in LOXHD1 short isoform that has not been characterized previously (Grillet et al., 2009). Coiled-coil domain-containing proteins are associated with critical biological functions such as transcription and cell movement. Notable examples are the transcription factor c-Fos and c-Jun, as well as the muscle protein tropomyosin (Truebestein and Leonard, 2016). Here, the identification of NLS near the coiled-coil domain in LOXHD1, its localization to the nucleus, its effect on EWSR1::FLI1 transcriptional program, and its potential role in HIF1α stability under hypoxia suggests a direct role of this enigmatic protein as a mediator of oncogenic functions in EwS cells.

Tumor hypoxia is a common feature of solid malignancies, including EwS, and HIF1α along with HIF2α are primary transcription factors required for cellular adaptation to hypoxia that significantly influence metastatic potential of tumor cells (Aryee et al., 2010; Semenza, 2012). Under hypoxia, HIF1α is upregulated through protein stabilization via functional inactivation of the Elongin B/C-CUL2-VHL E3 ligase complex, known to target HIF1α for proteasomal degradation (Jaakkola et al., 2001). Studies have identified few critical molecules in the HIF1α pathway in EwS, such as YB-1 as a direct HIF1A translation regulator and EWSR1::FLI1 as the downstream HIF1α target (Aryee et al., 2010; El-Naggar et al., 2015). However, factors influencing the stabilization of HIF1α in the context of EwS are not well understood. Our transcriptional and protein data suggest LOXHD1 as a new player in cellular adaptation to hypoxia through its ability to stabilize HIF1α in EwS. LOXHD1 silencing led to an attenuated hypoxic transcriptional program that substantially impaired EwS cell metastasis. In addition, LOXHD1 was found to interact with HIF1α, and silencing LOXHD1 affected HIF1α at the protein level. The data together suggest that in EwS, LOXHD1 directly interacts with HIF1α in the nucleus and protects it from proteasomal degradation. Further research is needed to delineate the molecular mechanism of LOXHD1-mediated hypoxic stress response via HIF1α stabilization, cytoskeleton organization, and transcriptional regulation through direct interaction with DNA/transcription factors/chromatin in EwS cells.

Immunotherapy has emerged as the next frontier in cancer treatment (Mellman et al., 2011). Adoptive cell therapy (ACT) with T cells engineered to recognize non-mutated tumor-associated antigens offers an attractive alternative. This is supported by encouraging clinical trial results with TCR gene therapy directed against the NY-ESO-1 tumor antigen in patients with synovial sarcoma and metastatic melanoma demonstrating a durable complete cancer regression (D’Angelo et al., 2018; Faramarzi and Ghafouri-Fard, 2017). These results have stimulated efforts to genetically modify lymphocytes to improve their specific antitumor efficacy and to extend the range of tumors that can be targeted. However, a significant impediment to the development of effective immune-based therapies for EwS is in identifying tumor-specific molecules with a limited expression in healthy tissues. Ideally, the target antigen must be derived from a protein that is (1) highly expressed in tumor cells (to ensure on-target activity), (2) minimally expressed in normal tissue (to reduce off-target activity/toxicity), and (3) required for tumor cell survival/sustenance (to prevent therapy resistance) (June et al., 2015; Roberts and Mullighan, 2015; Sotillo et al., 2015). Our observations demonstrating the highly exclusive expression pattern of LOXHD1, and functional validation of its oncogenic potential, fulfill these criteria for a potential ACT-based immunotherapy against LOXHD1 in EwS.

In summary, our findings identify LOXHD1, which is transcriptionally silent in the vast majority of normal and cancer cells, as a direct EWSR1::FLI1 target gene that plays an important role in cytoskeletal homeostasis, hypoxic adaptation, and oncogenic transcription in EwS. We show that loss of LOXHD1 short-isoform expression through deletion or epigenetic silencing of EWSR1::FLI1 bound *de novo* enhancer strongly inhibits the tumorigenic potential of EwS cells *in vitro* and *in vivo*. While more functional characterization of LOXHD1 needs to be made, we believe this study provides a strong basis for using LOXHD1 as a diagnostic/prognostic marker and identifying LOXHD1-derived endogenous peptide epitopes in EwS cells for ACT-based immunotherapy for this deadly disease.

### Limitations of the study

While we identified the ESS32 gene signature for EwS stratification and demonstrated LOXHD1 as an EWSR1::FLI1-regulated oncogene in EwS, the study has limitations. First, since EwS is a rare disease, there is a shortage of high-quality expression datasets for ESS32 validation. Second, the rarity of fresh tumor specimens also hindered us from testing the role of LOXHD1 in patient-derived tumor models. Third, mechanistically, the nuclear function of LOXHD1 is not thoroughly investigated here, and it remains unclear what role it may have other than modulating HIF1α and EWSR1::FLI1 pathways. Last, it remains to be determined whether LOXHD1 activation alone is sufficient to drive EwS metastasis.

## STAR★METHODS

### RESOURCE AVAILABILITY

#### Lead contact

Further information and requests for resources and reagents should be directed to and will be fulfilled by the lead contact, Irfan A. Asangani (asangani@upenn.edu).

#### Materials availability

Plasmids generated in this study can be requested and fulfilled by the lead contact, Irfan A. Asangani (asangani@upenn.edu).

#### Data and code availability

The RNA-seq and ChIP-seq data have been deposited in NCBI’s Gene Expression Omnibus under accession number accession number GSE163335.

This paper does not report an original code.

Any additional information required to reanalyze the data reported in this paper is available from the lead contact upon request.

### EXPERIMENTAL MODEL AND SUBJECT DETAILS

#### Cell lines

Human EwS cell lines RD-ES (obtained from CLS), SK-N-MC, CHLA-10, CADO-ES1, prostate cancer cell lines LNCaP, 22RV1, and osteosarcoma U2OS cell lines (obtained from ATCC) were maintained in RPMI-1640 media (Gibco, 11875093) supplemented with 10% FBS (HYC, SH30910.03) and 1% penicillin-streptomycin (Invitrogen, 15140122). All cells were grown at 37°C in a 5% CO_2_ incubator. For the hypoxia experiment, the cells were incubated in hypoxia chamber of 1% of O_2_, 5% CO_2_ at 37°C for the indicated time. All cell lines were tested negative for mycoplasma contamination and authenticated by STR profiling.

#### Chicken chorioallantoic membrane assay for tumor cell intravasation

The CAM assay for tumor cell intravasation was performed as previously described (Asangani et al., 2013). Briefly, fertilized chicken eggs were incubated in a rotary humidified incubator at 38°C for 10 days. A small hole was drilled through the eggshell into the air sac and another hole was drilled near the allantoic vein that penetrates the eggshell, keeping the chick chorioallantoic membrane (CAM) intact. The CAM was dropped by applying mild vacuum to the hole over the air sac. Subsequently, a cutoff wheel (Dremel) was used to cut a 1 cm^2^ window encompassing the second hole near the allantoic vein to expose the underlying CAM. Cells were prepared for implantation by trypsinizing and resuspending in media (without FBS) at the density of 2 × 10^6^ cells/50 μL. The CAM was gently abraded with a sterile cotton swab to provide access to the mesenchyme and 50 μL cell suspension was implanted on top of it. The windows were sealed, and the eggs returned to a stationary incubator. The eggs remained in the incubator for 72h, after which the egg was cut along the long circumference, and the upper half (with the inoculum) and the content of the egg was discarded; the CAM that lines the cavity of the eggshell was lifted and snap-frozen. Genomic DNA from the lower CAM was extracted using PureGene Genomic DNA isolation kit (Gentra-Qiagen) following the manufacturer’s protocol and used as a template for human Alu sequence amplification by q-PCR to quantitate the difference in the number of cells between control group and LOXHD1 KD group.

#### Zebrafish migration assay

All procedures on zebrafish (Danio rerio) were approved by the Institutional Animal Care and Use Committee of the University of Pennsylvania. Fertilized zebrafish eggs were incubated at 28°C in E3 solution and raised using standard methods. Embryos were transferred to E3 solution containing 5 μg/mL protease and 0.2 mM1-phenyl-2-thio-urea (PTU, Sigma) at 24 h post-fertilization to dechorionate the fish embryos and prevent pigmentation, respectively. At 48 h post-fertilization, zebrafish embryos were anesthetized with 0.03% tricaine (Sigma) and transferred to an injection plate made with 1.5% agarose gel for microinjection. Approximately 200–400 mCherry tagged EwS cells suspended in conditioned media supplemented with 0.5 mM EDTA were injected into the perivitelline space of each embryo using a XenoWorks Digital Microinjector (Sutter Instrument). Pre-pulled micropipettes were used for the microinjection (Tip ID 50 μm, base OD 1 mm, Fivephoton Biochemicals). After injection, the fish embryos were immediately transferred to PTU-E3 solution. Injected embryos were kept at 33°C and examined every day to monitor tumor cell migration using an Olympus Ix81 widefield microscope.

#### EwS mouse xenograft assay

Animal experiments were approved by local authorities and conducted in accordance with ARRIVE guidelines, recommendations of the European Community (86/609/EEC), and UKCCCR (guidelines for the welfare and use of animals in cancer research). 10–12 weeks old NOD/Scid/gamma (NSG) both female and male mice were used for Ewing sarcoma xenograft assay. 3.5×10^6^ SK-N-MC EwS cells were injected in a 1:1 mix of cells suspended in PBS with Geltrex Basement Membrane Mix (Thermo Fisher) in the right flank of the mice. Tumor diameters were measured every second day with a caliper and tumor volume was calculated by the formula L×l^2^/2. Once the first tumor of the control group reached an average volume of 1,500 mm^3^, all animals of the experiment were sacrificed by cervical dislocation. Other humane endpoints were determined as follows: Ulcerated tumors, loss of 20% body weight, constant curved or crouched body posture, bloody diarrhea or rectal prolapse, abnormal breathing, severe dehydration, visible abdominal distention, obese Body Condition Scores (BCS), apathy, and self-isolation.

### METHOD DETAILS

#### Plasmids

To generate pcDNA3-LOXHD1-Myc : Full Loxhd1 CDS was cloned from P7 mouse organ of Corti using the following primers: NG-138 and NG-64, then re-amplified using primers containing XhoI and EcorI sites respectively and cloned into pcDNA3. Myc tag was added by using NEBuilder Hifi 2X mixture with primers: PW-83 and PW-84 (Table S6). To generate NLS-coiled-coil-HA constructs, we amplified the sequence of NLS-Coiled-coil from adult mouse testis cDNA, then ligated to pcDNA3.1(+) vector. NheI and NotI sites were used for the construction. HA tag, NheI and NotI sites were added by PCR primers: NG-191-NheI-CC-S and NG-192-NotI-CC-AS (Table S6). pCDNA-HA-HIF1A was a gift from Dr. Frank S. Lee at UPENN, PA.

#### 5′ RACE

To identify the transcriptional start site of the LOXHD1 short isoform, 5′ RACE (Rapid Amplification of cDNA Ends) was performed using the Invitrogen 5’ RACE kit (Cat. NO. 18374–058) as described by the manufacturer’s protocol. The 5′ RACE-ready first-strand cDNA was synthesized using total RNA from SK-N-MC, RD-ES and CADO-ES1 cells. Briefly, the first strand cDNA was synthesized from the total RNA using LOXHD1 gene specific primer 1 (GSP1) and separated using a S.N.A.P. column. A homopolymeric tail is then added to the 3′ end of the cDNA followed by PCR amplification using LOXHD1 GSP2 and GSP3 (see Table S6 for primer sequence information) RACE PCR products were separated on a 1.5% agarose gel. Gel products were extracted with a Gel Extraction kit (Qiagen), cloned into pCR-TOPO vectors and sequenced at the University of Pennsylvania Sequencing Core. At least three colonies were sequenced for every RACE PCR product that was gel purified.

#### CRISPR-cas9 mediated LOXHD1 enhancer knockout and enhancer silencing

sgRNAs targeting adjacent to the LOXHD1 GGAA microsatellites were designed with the following website: https://portals.broadinstitute.org/gpp/public/analysis-tools/sgrna-design. For the enhancer region knockout, we inserted two pairs of sgRNAs into lentiCRISPR v2 (Plasmid #52961) backbone. For LOXHD1 repression, two independent sgRNAs were cloned into a lentiviral backbone expressing sgRNA and dCas9-KRAB (Plasmid #71236). Lentivirus was packaged using 2nd generation lentiviral packaging systems using the following protocol. 1×10^6^ HEK-293HT cells were seeded in 10cm plates. Next day lentiCRISPR or lenti-dCas9-KRAB plasmids (4 μg) were co-transfected with pVSVg (1 μg) and pSPAX2 (2 μg) using 7 μL Lipofectamine 2000. Media was collected after 48h and 72h of transfection, centrifuged and passed through 0.45 μm filters to clear of any live cells or debris. Virus were then concentrated by 10X with LentiX concentrator (Takara, 631232) aliquoted and stored at −80°C. RD-ES and SK-N-MC cells were infected at a MOI of 10. We selected for positive cells with puromycin at 1μg/ml for three days to obtain stably knock-out/down cells. Cells within 5 passages after puromycin selection were used for all the experiments.

#### shRNA knockdown

shRNAs against FLI1, and ERG (Penn Core Facilities) were packaged using second-generation lentiviral packaging systems as described above. CHLA-10, RD-ES and SK-N-MC were transduced with shFLI1 lentivirus and CADO-ES cells were transduced with shERG lentivirus. Four days post infection, cells were harvested for qRT-PCR analysis.

#### U2OS EWSR1::FLI1 overexpression

EWSR1::FLI1 virus was packaged using pMSCV-Hygro-EWSr1::FLI1, pGag-Pol and pVSV-G in HEK293T cells. U2OS cells were infected with the fusion virus for two days followed by two days of Hygromycin 200μg/ml selection. The cells then were subjected for RNA extraction and qPCR analysis using LOXHD1 and EWSR1::FLI1 primers as described below. Meanwhile the fusion overexpressed cells were also used for ChIP qPCR analysis as described below.

#### RNA extraction and quantitative RT-PCR

Total RNA was isolated from cells using the miRNeasy kit (QIAGEN, 217004) and cDNA was synthesized from 1,000 ng total RNA using SuperScript IV First-Strand Synthesis SuperMix (Life Technologies, 18091200). qPCR was performed using Fast SYBRgreen (Life Technologies, 4385612) on a StepOnePlus Real-Time PCR system (Applied Biosystems). Relative expression was calculated using ΔΔCT values normalized against *GAPDH* expression. All primers were designed using primer 3 (http://frodo.wi.mit.edu/primer3/) and synthesized by Integrated DNA Technologies.

#### Chromatin immunoprecipitation (ChIP) qPCR and ChIP sequencing

ChIP was performed using iDeal ChIP-seq Kit for Transcription Factors (Diagenode, C01010170) according to manufacturer’s protocol. In brief, SK-N-MC and RD-ES cells of the control and knockdown groups were trypsinized, washed, and crosslinked with 1% formaldehyde in culture medium for 10 min at room temperature. Cross-linking was terminated by the addition of 1/10 volume 1.25 M glycine for 5 min at room temperature followed by cell lysis and sonication (Bioruptor, Diagenode), resulting in an average chromatin fragment size of 200bp. Chromatin equivalent to 5×10^6^ cells was isolated and incubated with 5 μg antibody overnight at 4°C (H3-acetyl K27, H3K4me3, H3K9me3, FLI1 and IgG (Diagenode). ChIP DNA was isolated by washing and reversal of cross-linking. The eluted DNA was used for SYBRgreen qPCR. The primer sequences used for ChIP-qPCR are provided in Table S6.

10ng of the ChIP DNA was used for ChIP sequencing library preparation following the protocol of TruSeq ChIP library preparation kit (Illumina, IP-202-1012). Briefly, A single “A” nucleotide was added to the 3′ ends of the blunt-ended ChIP DNA fragments and then ligated to a unique adapter. The ligation products were purified and selected at the size of 250–300bp by 4% NuSieve agarose gel (Lonza) electrophoresis. Size selected DNA was purified and PCR-amplified and quantitated with the Bioanalyzer 2100 (Agilent). Libraries were pooled and ran on Nextseq500 platform (Illumina Inc.) with high throughput single-end reads of 75bases (Cat.TG-160-2005).

#### Datasets used in meta-analysis for the computational pipeline

Our computational pipeline to identify EWSR1 FLI1-regulated genes used an integrative analysis of various RNA and ChIP-seq datasets sourced from publicly curated repositories and from datasets published in Gene Expression Omnibus (GEO). Details of the datasets used in our analysis are shown in Tables S1 and S2.

#### Network plots for correlation of ESS32 expression in MET500 + EwS datasets

We computed the Pearson correlation coefficient (*r*) for ESS32 expression in the MET500 + EwS datasets using a custom in-house python script. We used the coefficients to generate a graph network (Figure 1D) in which each sample was represented as a node, colored by sample cohort, and the connectivity between any two nodes being proportional to *r*. Connectivity between nodes with *r* < 0.5 are not shown for clarity. The graph network was represented using the graphml format and rendered using gephi (https://gephi.org/).

#### ChIP-seq analysis

Sequencing reads for both in-house generated and publicly available datasets were uniformly processed using an in-house ChIP-seq analysis pipeline. Reads were quality checked with FASTQC (www.bioinformatics.babraham.ac.uk/projects/fastqc) and aligned to the GRCh37 (release 27) genome using the STAR v2.5.1 aligner (Dobin et al., 2013) with default settings. PCR duplicate reads in the aligned bam files were removed using samtools (Li et al., 2009), bam files were converted to CPM normalized bigwig tracks using deeptools (Ramirez et al., 2016), and viewed using the Integrated Genomics Viewer or the UCSC genome browser. Single end reads were extended up to the fragment length (200bp) along the read direction.

#### Enrichment analysis of ChIP-seq data

The enrichment peaks for the various transcription factors and histone marks were computed using MACS2 (Zhang et al., 2008), with default settings. Regions found to ubiquitously enriched across a number of next-generation sequencing experiments (ENCODE Project Consortium, 2012), also known as the blacklisted peaks (https://sites.google.com/site/anshulkundaje/projects/blacklists), were excluded in all subsequent analysis.

#### Overlap of enrichment peaks

Overlap analysis of enrichment peaks in different samples was performed using an in-house python script. In our analysis, two peaks were taken to be overlapping even if they were a single base.

#### RNA-seq library preparation and sequencing

After the treatments, total RNA was isolated using miRNeasy kit (QIAGEN) and the quality of the RNA was analyzed by Bio-analyzer (Agilent) using RNA nano chip. After confirming that all of the RNA samples have RNA integrity number (RIN) more than 8, RNA-seq libraries were constructed using the TruSeq RNA Library Prep Kit v2 (Illumina, RS-122-2001) according to the manufacturer’s instructions. Briefly, 1 μg of purified RNA was poly-A selected and fragmented with fragmentation enzyme. After first and second strand synthesis from a template of poly-A selected/fragmented RNA, other procedures from end-repair to PCR amplification were performed according to the instructions given in the protocol. Libraries were purified and validated for appropriate size on a 2100 Bio-analyzer DNA 1000 chip (Agilent Technologies, Inc.). The DNA library was quantitated using Qubit and normalized to 4 nM prior to pooling. Libraries were pooled in an equimolar fashion and diluted to a final concentration of 1.8 pM. Library pools were clustered and run on Nextseq500 platform (Illumina Inc.) with single-end reads of 75 bases (Cat.TG-160-2005).

#### RNA-seq analysis

Single-end sequencing reads were demultiplexed using Illumina *bcl2fastq*, quality checked using FASTQC (www.bioinformatics.babraham.ac.uk/projects/fastqc), and aligned to the GRCh37 (release 27) genome using the STAR v2.5.1 aligner (Dobin et al., 2013), with default settings. The read statistics generated by STAR v2.5.1 was used to ensure that the aligned reads in all samples were over 90% of the total reads. The transcripts were assembled using cufflinks and the count and FPKM tables were computed using cuffnorm (Trapnell et al., 2010). Principal component analysis on the FPKM values was used to cluster the samples for further quality check.

#### Differential gene expression analysis

Genes differentially expressed between any two sets of treatment conditions were determined using DESeq2 (https://bioconductor.org/packages/release/bioc/html/DESeq.html), a statistical tool that employs shrinkage estimates to compute fold changes. In all our calculations, the raw RNA-seq read counts from biological duplicates, for each treatment condition, was used as the input for DESeq2. Heatmaps for differentially expressed genes were generated using in-house python scripts.

#### Gene Ontology analysis

We determined the Gene Ontology (GO) associated with a given set of genes using the python api for g:Profiler (https://biit.cs.ut.ee/gprofiler). The results were plotted using in-house python scripts.

#### Gene set enrichment analysis (GSEA)

Functional class scoring of the all differentially expressed genes, against a given gene signature, was performed using the GSEA tool (Subramanian et al., 2005), developed by the Broad Institute (https://software.broadinstitute.org/cancer/software/gsea/wiki/index.php/Main_Page).

#### Immunoblot analysis

For immunoblot analyses, cells were lysed in RIPA buffer (Boston Bioproducts, BP-115DG) supplemented with protease inhibitor (Pierce, A32965). Samples for HIF1α immunoblot were harvested in 1x Laemmli sample buffer (Bio-Rad, 1610737). NE-PER™ Nuclear and Cytoplasmic Extraction Reagents (Thermo Fisher) were used for cytoplasmic and nuclear fractionation, Lysates were boiled in SDS sample buffer (Invitrogen) and 30–50 μg of protein was separated by SDS-PAGE and loaded onto a PVDF membrane. Membranes were blocked for one hour in blocking buffer (Tris-buffered saline, 0.1% Tween (TBS-T), 5% non-fat dry milk) and incubated overnight at 4°C in primary antibody. Blots were washed with TBS-T and incubated with HRP-conjugated secondary antibody for one hour at room temperature. Blots were washed again with TBS-T and visualized after incubation with chemiluminescent substrate (GE Healthcare). The antibodies used in the study are provided in the key resources table.

#### Immunoprecipitation

For the immunoprecipitation assay with the MYC tagged LOXHD1, HEK293T cells were transfected with pCDNA-HA-HIF1α (gift from Dr. Frank S. Lee, UPENN) and pCDNA-Myc-LOXHD1 at a 1 to 2 ratio. 48h post-transfection, cells were incubated in 1% of O_2_ hypoxia chamber for 6h followed by lysis in IP buffer (20 mM Tris pH7.5, 150 mM NaCl, 1% Triton-X 100, Protease Inhibitor) and sonication. Whole cell lysates (500 μg) were pre-cleaned by incubation with protein G Dynabeads (Life Technologies) for 2h on a rotator at 4°C. 5 μg antibody was added to the pre-cleared lysates and incubated on a rotator at 4°C overnight. Protein G Dynabeads were then added for 2h. Beads were washed four times in IP buffer, containing 300 mM NaCl, and resuspended in 40 μL of 1x Laemmli sample buffer and boiled at 95°C for 5 min for separation of the protein and beads. Samples were then analyzed by SDS-PAGE and western blotting as described above.

For the immunoprecipitation assay with the endogenous HIF1α, SK-N-MC cells were incubated in 1% O_2_ hypoxia chamber for 16hrs to stabilize HIF1α. Nuclear lysate was extracted using NE-PER™ Nuclear and Cytoplasmic Extraction Reagents (Thermo Fisher), IP was immediately proceeded using the same protocol described above with 900 μg of the nuclear lysate and 10 μL of the HIF1α antibody.

#### Immunofluorescent staining

For general staining 100,000 cells were seeded on a coverslip which fits in wells of 24-well plate, the next day, cells were fixed with 4% of paraformaldehyde for 10min at RT. Cells were then washed with PBS and blocked with 10% goat serum and 0.5% Triton 100 in PBS at RT for 60min. Primary antibody of LOXHD1 and/or HA was diluted at 1:500 in 10% of goat serum in PBS and incubated with the cells at 4°C overnight. On the next day, secondary antibody goat anti-Rabbit Alexa Fluor 568 (Thermo Fisher, A-11011) and/or anti-Mouse Alexa Fluor 488 (Thermo Fisher, A28175) was diluted at 1:1000 and incubated at RT for one hour. Coverslips were then mounted and stained for DAPI with anti-fade mounting medium (Vector, H-1200). Images were acquired and processed on a Zeiss confocal microscope (LSM 880).

For F-actin staining, cells were plated in the same way, stained with Phalloidin-iFluor 488 at 1:1000 at room temperature for 1h. Images were acquired and processed on a Zeiss confocal microscope (LSM 880). Cell surface area were measured with ImageJ.

For HA-NLS-coiled coil staining in 293T cells: 293T cells (ATCC, CRL-3216) were plated at a density of 8×10^4^ in per well of a 24-well plate with Ploy-L-Lysine (Sigma-Aldrich, P8920-100ML) coated cover slips the day before transfection. 500ng plasmids were transfected by using FuGENE HD regent. 24h and 48h after transfection, the cells were fixed with 4% PFA for 10 min, RT. The cells were permeabilized and blocked by incubation in blocking buffer (4% BSA, 0.5% saponin in PBS) for 1h at room temperature and then incubated with 1^st^ antibody (rat anti-HA, 3F10, Roche, 1:200 in blocking buffer) overnight at 4°C. The next day, the cover slips were washed with PBS for three times and then incubated in 2^nd^ antibody (Alexa Fluor 488, Goat anti-rat IgG, Thermo Fisher, A11006, 1:300) and DAPI (Sigma-Aldrich, D8417, 30ng/ml) diluted with blocking buffer for one hour at room temperature, followed by 3 times of washing with PBS. Lastly, coverslips were mounted with Prolong Gold anti-fade mounting medium (Invitrogen, P36934). The images were acquired using Zeiss LSM 880 confocal microscope.

#### Colony and sphere formation assay

For colony formation assay, 5,000 cells were plated in one well of the 6-well plates, in two weeks cells were fixed and stained with 0.5% of crystal violet, quantification was done by de-staining of crystal violet and measure absorbance at 560nm. For sphere formation assay, 500 cells were suspended in 100μL 50% of Matrigel of full RPMI culture media and spread on the edge of wells in a 24-well plate, we then fill the wells with 1 mL of full RPMI culture media. In three weeks, spheres were counted, and the sizes of the spheres were measured with ImageJ. Triplicates were carried in all the above described experiments; student t-test was used for statistical analysis.

#### Cell aggregation assay

24-well plates were coated with 100 μL of 3% of poly-HEMA and dried in cell culturing hood overnight. 10,000 cells in single cell suspension were seeded in the wells, pictures were taken at time 0 and 16hr after seeding. Aggregation sizes were measured with ImageJ, student t-test was used for statistical analysis.

#### Wound healing assay

Cells were grown as monolayer in 6-well plate, scratches were made with 1000 μL tips. Pictures of the same area were taken at 24 and 48h. Gap between scratches were measured by ImageJ.

#### Matrigel invasion assay

Stably knockout/down cells were trypsinized and 100,000 cells were suspended in 500μL serum-free RPMI medium and added into Matrigel coated invasion chambers (Corning, 354480). Groups of hypoxic and normoxic samples were performed side by side. The bottom of the chamber was filled with RPMI containing 20% serum as chemo attractant. Cells that had degraded the matrix and migrated through the porous membrane (8 μm pore size) to the other end after a period of 24h or 48h were fixed and stained with crystal violet (0.5%), and images were captured using phase contrast microscopy. The same cells were seeded into chambers with no Matrigel coating as migration control. % invasion was determined by numbers of cells invaded divided by numbers of cells migrated. Invasion index was calculated by % invasion test cells / % invasion control cells. Triplicates were carried in all the above described experiments; we take six images for each well and count the cell numbers. Student t-test was used fonr statistical analysis.

#### Cell proliferation growth curve

100,000 of each indicated control and knockdown cells were seeded in 24 well plates on day 0, triplicate wells of each group were trypsinized and the cell numbers were counted using Countess II Automated Cell Counter on each indicated timepoint. The regular cultured experiments were performed for day 0, 2, 4 and 6. The hypoxic experiment was performed in 3% O_2_ for day 0, 2 and 4.

#### Cell cycle analysis

Control and eKD cells after five-day culture were trypsinized and washed with 1x PBS followed by fixation with ice-cold 70% ethanol suspended in PBS in a drop-wise manner and stored at −20°C for overnight. On the following day, cells were collected by centrifugation and washed with cold PBS for 3 times and resuspended in PBS containing 0.1% Triton X-100, 50 μg/mL Propidium Iodide (PI), and 500 μg/mL RNase A and incubated for 30 min at room temperature. Flow cytometry was executed on a FACSCalibur (BD Biosciences) by gating for PI staining and analyzed by using FlowJo software.

### QUANTIFICATION AND STATISTICAL ANALYSIS

Statistical analysis was performed using GraphPad Prism 6 software. For individual comparisons, paired or unpaired Student t test was used and p < 0.05 were considered significant.

## Supplementary Material

1

## Figures and Tables

**Table:** 

REAGENT or RESOURCE	SOURCE	IDENTIFIER

Antibodies		

H3K27ac	Active motif	Cat# 39133; RRID: AB_2561016
H3K4me3	Millipore	Cat# 07-473; RRID: AB_1977252
H3K9me3	Abcam	Cat# ab8898; RRID: AB_306848
FLI-1	Abcam	Cat# ab15289; RRID: AB_301825
GAPDH-HRP	Cell Signaling Technology	Cat# 3683; RRID: AB_1642205
HIF1α	Cayman Chemical Company	Cat # 10006421; RRID: AB_409037
HA Tag	Cell Signaling Technology	Cat# 3724S; RRID: AB_1549585
Myc-tag	Cell Signaling Technology	Cat# 2276S; RRID: AB_331783
Phalloidin-iFluor 488 Reagent	Abcam	Cat# ab176753
LOXHD1	Grillet et al., 2009	N/A
Mouse IgG	Diagenode	Cat# K01641008
Rabbit IgG	Cell Signaling Technology	Cat# 2729S; RRID: AB_1031062
Goat anti mouse IgG 594	Fisher Scientific	Cat# A11032; RRID: AB_2534091
Goat anti rabbit IgG 488	Fisher Scientific	Cat# A11008; RRID: AB_143165
Goat anti mouse IgG 488	Fisher Scientific	Cat# A11029; RRID: AB_2534088
Goat anti rabbit IgG 568	Fisher Scientific	Cat# A11011; RRID: AB_143157
anti-Rabbit HRP	Kindle BioSciences	Cat# R1006; RRID: AB_2800464
anti-Mouse HRP	Kindle BioSciences	Cat# R1005; RRID: AB_2800463

Bacterial and virus strains		

lentiCRISPR v2	Addgene	52961
pLV hU6-sgRNA hUbC-dCas9-KRAB-T2a-Puro	Addgene	71236
pMSCV-Hygro-EWS/FLI1	Boone et al., 2021	N/A

Critical commercial assays		

miRNeasy kit	Qiagen	217004
iDeal ChIP-seq Kit for Transcription Factors	Diagenode	C01010170
TruSeq ChIP library preparation kit	Illumina	IP-202-1012
TruSeq RNA Library Prep Kit v2	Illumina	RS-122-2001
5’ RACE system	Invitrogen	18374-058
Corning BioCoat Matrigel Invasion Chambers	Corning	354480
Halt™ Protease and Phosphatase Inhibitor	Thermo Scientific	78443
Single-Use Cocktail		
Trans-Blot Turbo Transfer System	Bio-Rad	1704271
NE-PER™ Nuclear and Cytoplasmic	Thermo Scientific	78835
Extraction Reagents		

Deposited data		

RNAseq and ChIP seq data	This paper	GEO: GSE163335

Experimental models: Cell lines		

Human SK-N-MC	ATCC	HTB-10
Human RD-ES	Cell Line Service	330410
Human CHLA-10	Children’s Oncology Group	CHLA-10
Human CADO-ES1	Cell Line Service	300127
Human LNCaP	ATCC	CRL-1740
Human 22RV1	ATCC	CRL-2505
Human U2OS	ATCC	HTB-96
Human 293T	ATCC	CRL-3216

Experimental models: Organisms/strains		

Mouse: NOD.Cg-Prkdcscid Il2rgtm1Wjl/SzJ	Jackson Laboratory	005557
Specific Pathogen Free Fertile Eggs	Charles River	10100326
Zebrafish Danio rerio	UPENN	N/A

Oligonucleotides		

Table S6		

Recombinant DNA		

LOXHD1-Myc	Trouillet et al., 2021 J Neurosci	N/A
HA-HIF1α	Yu et al., 2001 Cancer Research	N/A
NLS-coiled-coil-HA	This study	N/A

Software and algorithms		

Prism	Graphpad	https://www.graphpad.com/scientific-software/prism/
ImageJ/Fiji	N/A	imagej.nih.gov/ij/download.html
FlowJo	Tree Star	https://www.flowjo.com
STAR Aligner	https://doi.org/10.1093/bioinformatics/bts635	http://code.google.com/p/rna-star/
Gephi		https://gephi.org/
UCSC genome browser	http://www.genome.org/cgi/content/abstract/12/6/996	https://genome.ucsc.edu/
IGV	http://www.genome.org/cgi/content/abstract/12/6/996	https://genome.ucsc.edu/
GSEA	https://www.pnas.org/content/102/43/15545	https://www.gsea-msigdb.org/gsea/index.jsp
Gprofiler	https://biit.cs.ut.ee/gprofiler/gost	https://pypi.org/project/gprofiler-official/

Other		

Nextseq 500	Illumina	SY-415-1001
Zeiss LSM 880 Confocal	Zeiss	LSM880

## Highlights

- ESS32 gene signature stratifies Ewing sarcoma from other cancers
- EWSR1::FLI1 activates LOXHD1 expression in Ewing sarcoma through a *de novo* enhancer
- LOXHD1 silencing impairs Ewing sarcoma tumorigenicity
- LOXHD1 silencing attenuates hypoxia response and suppresses metastasis

## INCLUSION AND DIVERSITY

One or more of the authors of this paper self-identifies as an underrepresented ethnic minority in science. One or more of the authors of this paper self-identifies as a member of the LGBTQ+ community.
