# Supplementary material for: Oncofusion-driven de novo enhancer assembly promotes malignancy in Ewing sarcoma via aberrant expression of the stereociliary protein LOXHD1
Source: Cell Rep. Author manuscript; Available in PMC 2022 Dec 2. (PMC9716578; doi:10.1016/j.celrep.2022.110971)
Supplement: 1 [file NIHMS1841174-supplement-1.pdf]

Cell Reports, Volume *39*

Supplemental information

Oncofusion-driven *de novo* enhancer assembly promotes malignancy in Ewing sarcoma via aberrant expression of the stereociliary protein LOXHD1

Qu Deng, Ramakrishnan Natesan, Florencia Cidre-Aranaz, Shehbeel Arif, Ying Liu, Reyaz ur Rasool, Pei Wang, Erick Mitchell-Velasquez, Chandan Kanta Das, Endrit Vinca, Zvi Cramer, Patrick J. Grohar, Margaret Chou, Chandan Kumar-Sinha, Kristy Weber, T.S. Karin Eisinger-Mathason, Nicolas Grillet, Thomas Grünewald, and Irfan A. Asangani

Figure S1

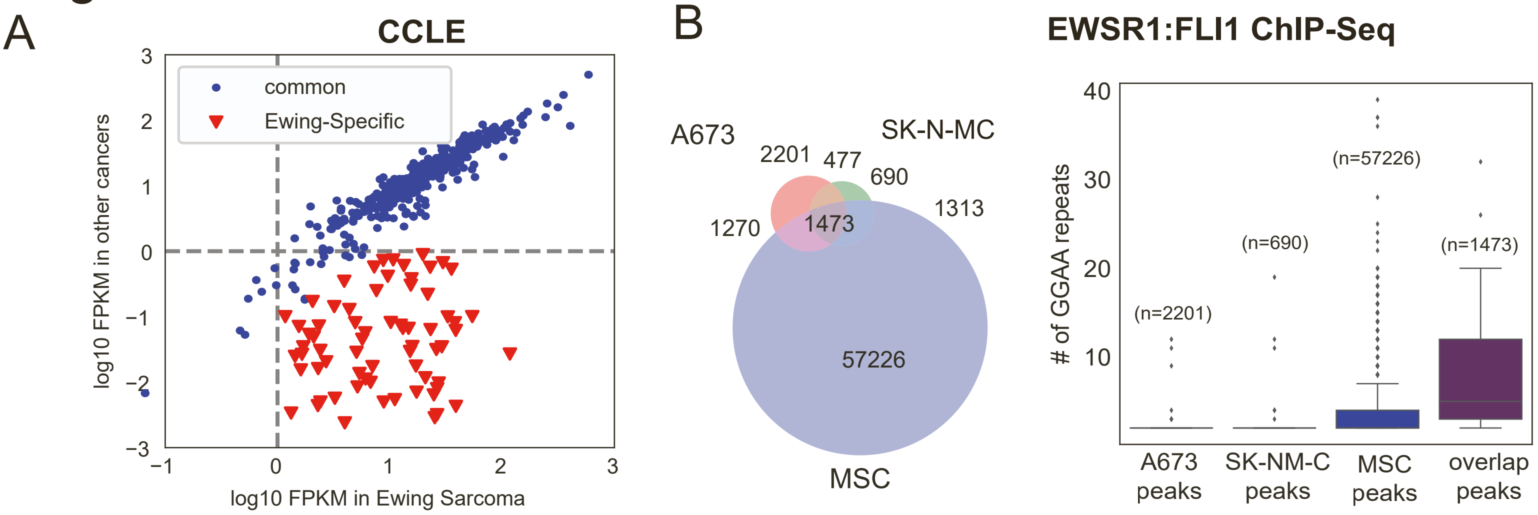

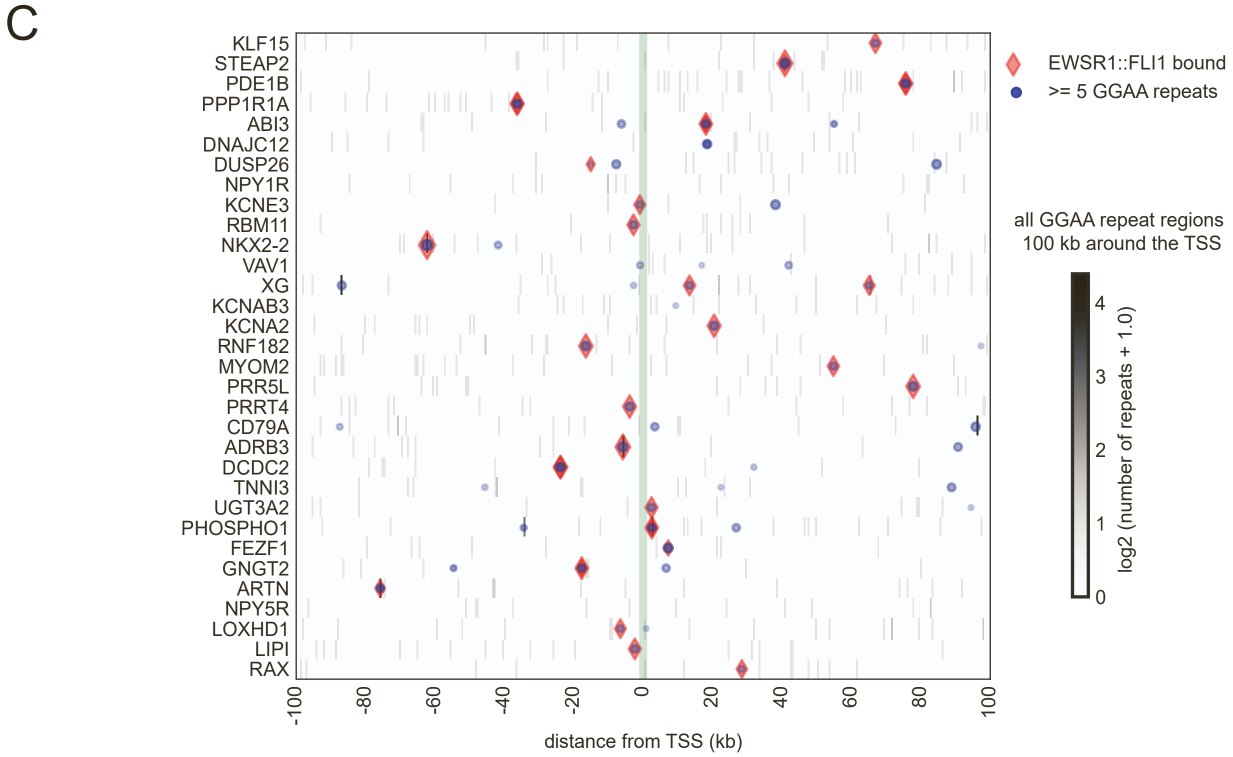

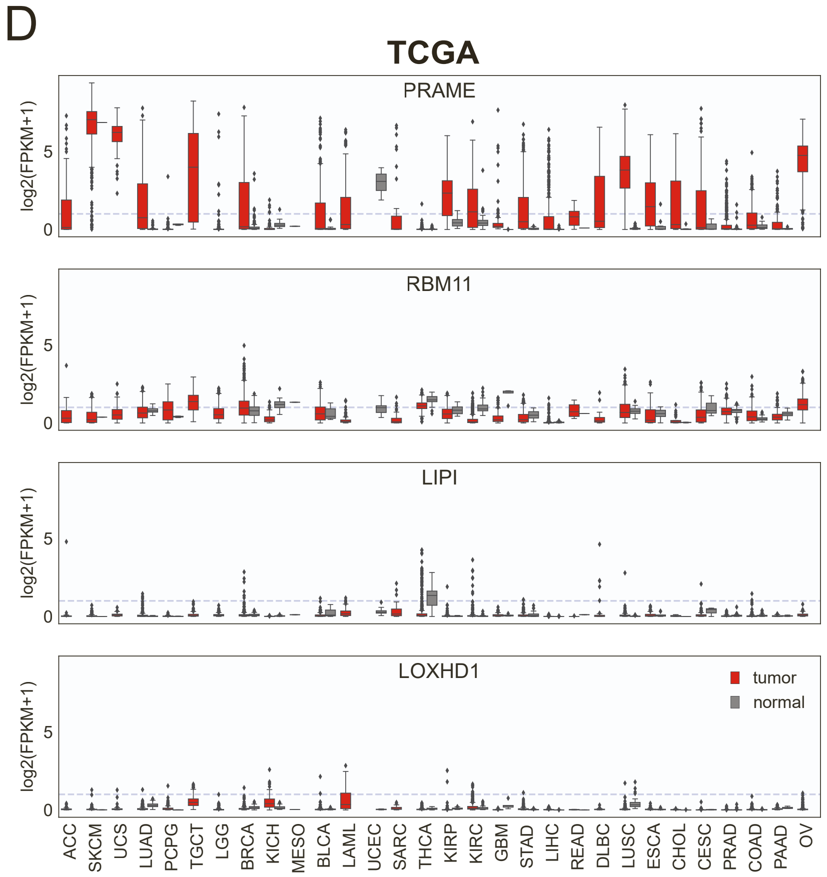

code	cancer	normal	tumor
LAML	Acute Myeloid Leukemia	0	151
ACC	Adrenocortical carcinoma	0	79
BLCA	Bladder Urothelial Carcinoma	19	411
LGG	Brain Lower Grade Glioma	0	529
COAD	Colon adenocarcinoma	41	464
ESCA	Esophageal carcinoma	11	162
GBM	Glioblastoma multiforme	5	168
KICH	Kidney Chromophobe	24	65
KIRP	Kidney renal papillary cell carcinoma	32	289
LIHC	Liver hepatocellular carcinoma	50	374
LUAD	Lung adenocarcinoma	59	526
LUSC	Lung squamous cell carcinoma	49	501
DLBC	Lymphoid Neoplasm Diffuse Large B-cell Lyr	0	42
MESO	Mesothelioma	0	1
OV	Ovarian serous cystadenocarcinoma	0	379
PAAD	Pancreatic adenocarcinoma	4	178
PCPG	Pheochromocytoma and Paraganglioma	3	150
PRAD	Prostate adenocarcinoma	52	499
READ	Rectum adenocarcinoma	1	6
SARC	Sarcoma	0	35
SKCM	Skin Cutaneous Melanoma	1	471
STAD	Stomach adenocarcinoma	32	375
TGCT	Testicular Germ Cell Tumors	0	156
THCA	Thyroid carcinoma	58	510
UCS	Uterine Carcinosarcoma	0	56

Figure S1: Integrative analysis of ChIP-seq and CCLE, MET500, TCGA and GTEx transcriptomic datasets (Related to Figure 1).

(A) *Analysis of CCLE dataset;* Expression of the 516 genes (step 1 of Fig. 1a) in EwS vs other cell lines. The 89 EwS specific genes, seen in the lower-right quadrant and encircled, are expressed only in EwS cell lines and show <1 FPKM expression in all others. **(B)** *Analysis of FLI1-ChIP-seq data; (left)* Overlap analysis between FLI1 enriched ChIP-Seq peaks in A673 (GSM1517562), SK-NM-C (GSM1517537), and EWSR1-FLI1 overexpressed MSCs (GSM2472088, GSM2472102, GSM2472108) yields 1473 conserved FLI1-bound regions, *(right)* distribution of the number of GGAA microsatellite repeats contained within A673-specific, SK-N-MC-specific, MSC-specific and the 1473 overlapping regions shows pronounced enrichment of the GGAA microsatellites in the overlapping regions. **(C)** Colormap shows the locations of GGAA microsatellite repeat regions ±100 kb around the TSS of the ESS32 genes. Symbol overlays represent regions with >= 5 GGAA repeats (blue circles) and contain a FLI1 ChIP-seq enrichment peak (red diamonds). **(E)** *Analysis of TCGA dataset;* Boxplot showing nearly zero expression of *LOXHD1* and *LIPI* in all TCGA cancer-subtypes in tumor and normal samples. The expression of *RBM11* located in *LIPI* locus is shown. Expression of PRAME is shown as an example of gene that is expressed across all cancer-subtypes. Abbreviation of codes for cancer-subtypes and the corresponding numbers of normal and tumor samples are shown alongside.

Figure S2

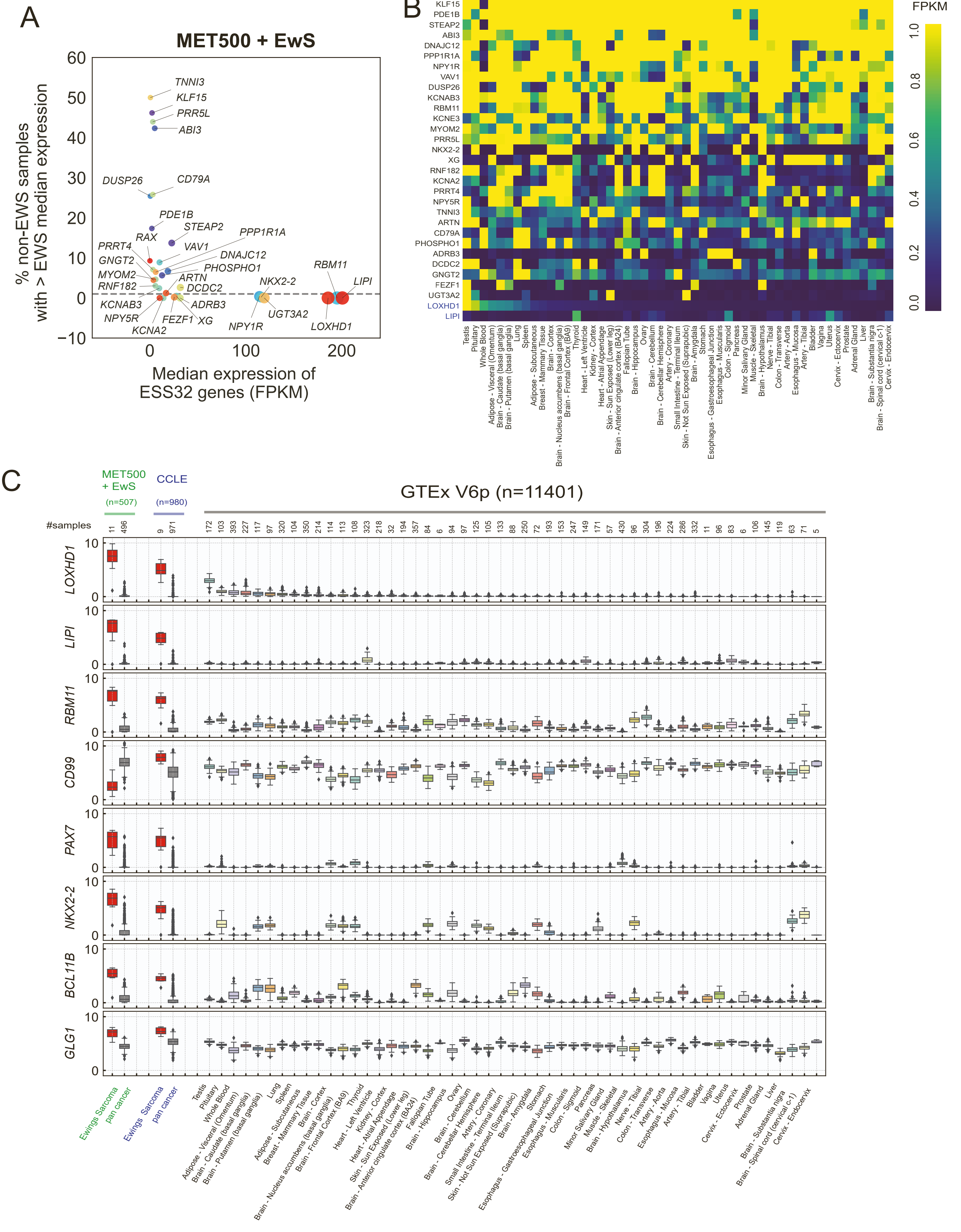

A
MET500 + EwS
% non-EWS samples with > EWS median expression
Median expression of ESS32 genes (FPKM)
TNNI3
KLF15
PRR5L
ABI3
DUSP26
CD79A
PDE1B
STEAP2
PPP1R1A
RAX
VAV1
PRRT4
DNAJC12
GNGT2
PHOSPHO1
MYOM2
ARTN
RNF182
DCDC2
KCNAB3
ADRB3
NPY5R
FEZF1
XG
KCNA2
NKX2-2
UGT3A2
NPY1R
RBM11
LIPI
LOXHD1
B
GTEx
FPKM
KLF15
PDE1B
STEAP2
ABI3
DNAJC12
PPP1R1A
NPY1R
VAV1
DUSP26
KCNAB3
RBM11
KCNE3
MYOM2
PRR5L
NKX2-2
XG
RNF182
KCNA2
PRRT4
NPY5R
TNNI3
ARTN
CD79A
PHOSPHO1
ADRB3
DCDC2
GNGT2
FEZF1
UGT3A2
LOXHD1
LIPI
C
MET500 + EwS
(n=507)
CCLE
(n=980)
GTEx V6p (n=11401)
#samples
LOXHD1
LIPI
RBM11
CD99
PAX7
NKX2-2
BCL11B
GLG1
Ewings Sarcoma
pan cancer
Ewings Sarcoma
pan cancer
Testis
Pituitary
Whole Blood
Adipose - Visceral (Omentum)
Brain - Caudate (basal ganglia)
Brain - Putamen (basal ganglia)
Lung
Spleen
Adipose - Subcutaneous
Breast - Mammary Tissue
Brain - Cortex
Brain - Nucleus accumbens (basal ganglia)
Brain - Frontal Cortex (BA9)
Thyroid
Heart - Left Ventricle
Kidney - Cortex
Heart - Atrial Appendage
Skin - Sun Exposed (Lower leg)
Brain - Anterior cingulate cortex (BA24)
Fallopian Tube
Brain - Hippocampus
Ovary
Brain - Cerebellum
Brain - Cerebellar Hemisphere
Artery - Coronary
Small Intestine - Terminal Ileum
Skin - Not Sun Exposed (Suprapubic)
Brain - Amygdala
Stomach
Esophagus - Gastroesophageal Junction
Esophagus - Muscularis
Colon - Sigmoid
Pancreas
Minor Salivary Gland
Muscle - Skeletal
Brain - Hypothalamus
Nerve - Tibial
Colon - Transverse
Artery - Aorta
Esophagus - Mucosa
Artery - Tibial
Bladder
Vagina
Uterus
Cervix - Ectocervix
Prostate
Adrenal Gland
Liver
Brain - Substantia nigra
Brain - Spinal cord (cervical c-1)
Cervix - Endocervix

Figure S2: Expression of ESS32 and known EwS diagnostic markers in MET500, CCLE, and GTEx transcriptomic datasets (Related to Figure 1).

(A) *Analysis of MET500+EwS RNA-seq dataset;* Plot showing the median expression of the ESS32 genes (step 3 in Figure 1A) in EwS against the percentage of non-Ewing samples that show expression higher than the median value in EwS. The dotted line marks the cutoff of 1%. **(B)** *Analysis of GTEx datasets;* Heatmap showing the expression of the ESS32 genes showing null expression of *LOXHD1*, *LIPI* in all tissues except testis. **(C)** Box plots comparing the expressions of indicated targets across MET500 + EwS (n=507), CCLE (n= 980), and GTEX (n=11401) samples.

Figure S3

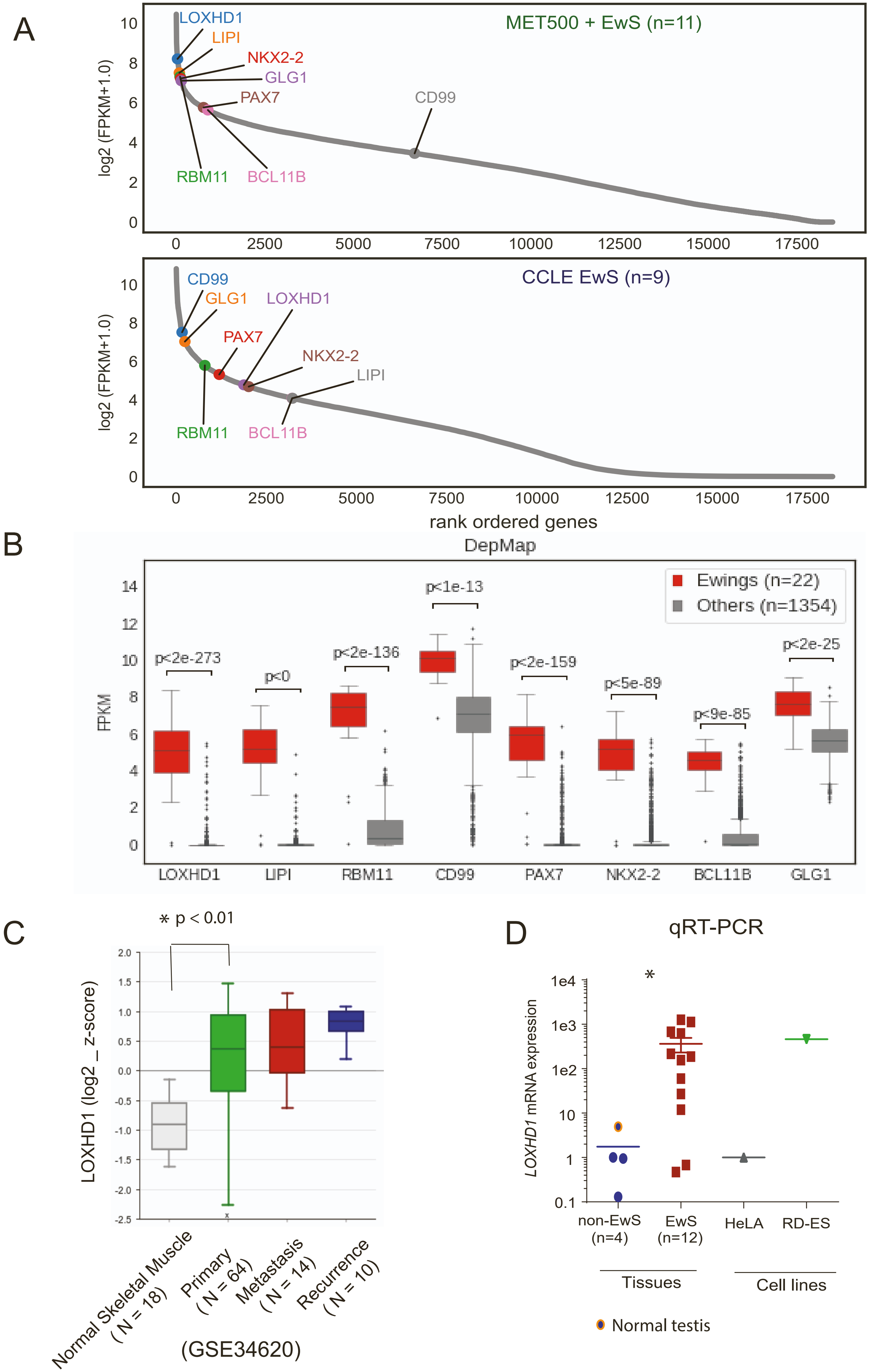
A
MET500 + EwS (n=11)
log2 (FPKM+1.0)
LOXHD1
LIPI
NKX2-2
GLG1
PAX7
CD99
RBM11
BCL11B
CCLE EwS (n=9)
rank ordered genes
B
DepMap
Ewings (n=22)
Others (n=1354)
FPKM
p<2e-273
p<0
p<2e-136
p<1e-13
p<2e-159
p<5e-89
p<9e-85
p<2e-25
LOXHD1 LIPI RBM11 CD99 PAX7 NKX2-2 BCL11B GLG1
C
* p < 0.01
LOXHD1 (log2 _ z-score)
Normal Skeletal Muscle (N = 18)
Primary (N = 64)
Metastasis (N = 14)
Recurrence (N = 10)
(GSE34620)
D
qRT-PCR
LOXHD1 mRNA expression
*
non-EwS (n=4)
EwS (n=12)
HeLA
RD-ES
Tissues
Cell lines
Normal testis

Figure S3: Ewing sarcoma specific LOXHD1 expression (Related to Figure 1).

(**A**) Shown are the rank ordered mean gene expression in EwS samples in the MET500+EwS and CCLE datasets. LOXHD1 and LIPI show consistently higher expression in the metastatic EwS samples. **(B)** mRNA FPKM expressions of n=1376 cell lines were obtained from the DepMap database. Box plots show expression of the marked genes in Ewing Sarcoma samples (n=22) versus all other cancers (n=1354). p-values displayed in the plot were computed using an independent Student's t-test. **(C)** Box plots showing LOXHD1 expression in the indicated samples. Publicly available Affymetrix dataset (GSE34620) comprising 117 EwS samples was analyzed and Log2 z-score is plotted. $p < 0.01$ One-way Anova between the groups. **(D)** qRT-PCR analysis of *LOXHD1* mRNA expression in an independent cohort of EwS tumor tissues, non-EwS tissues (testis is indicated with orange border), HeLa and RD-ES cells were used as negative and positive control, respectively. * $p < 0.001$, by two-tailed Student's t test.

Figure S4

A

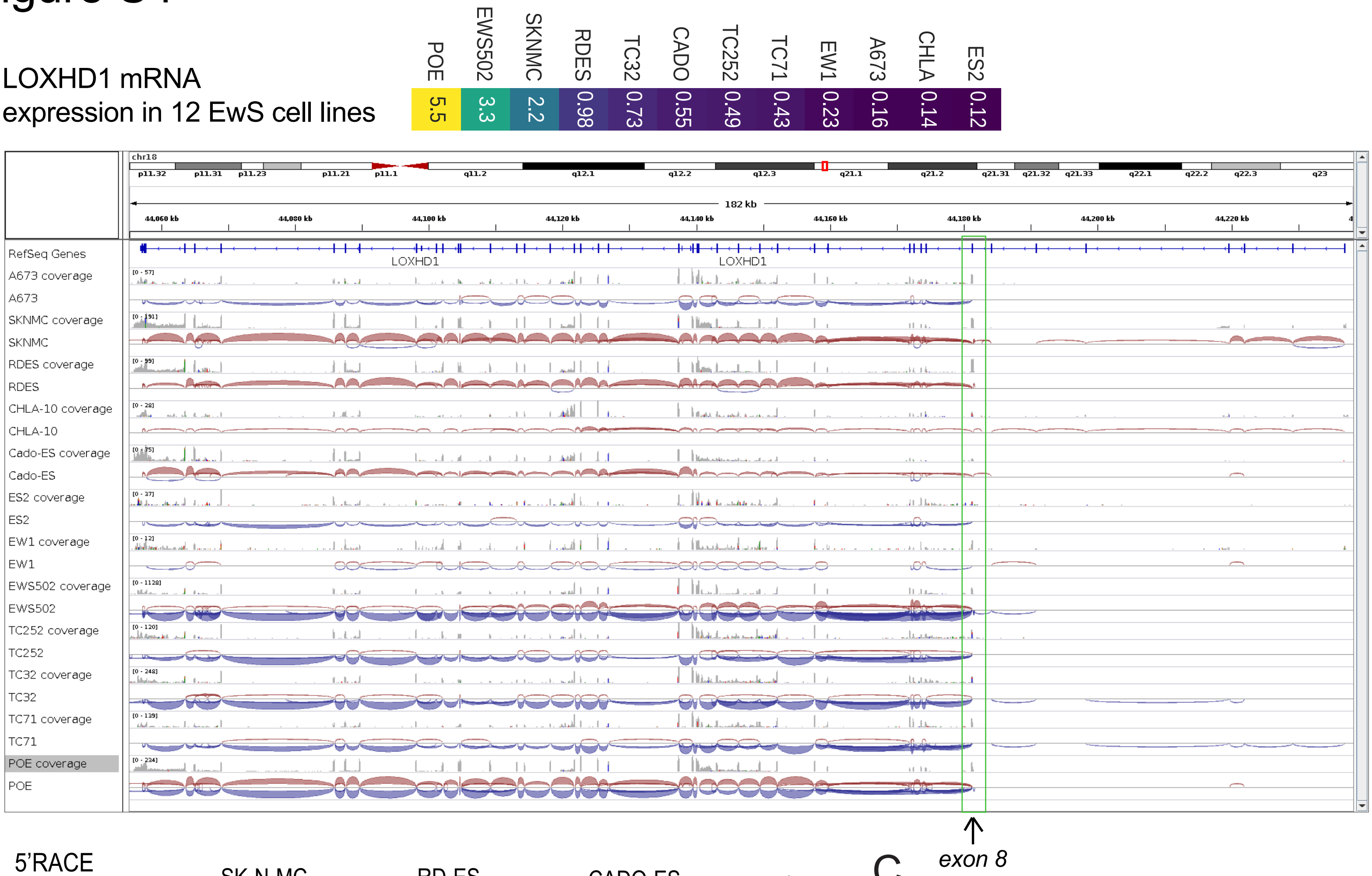

B

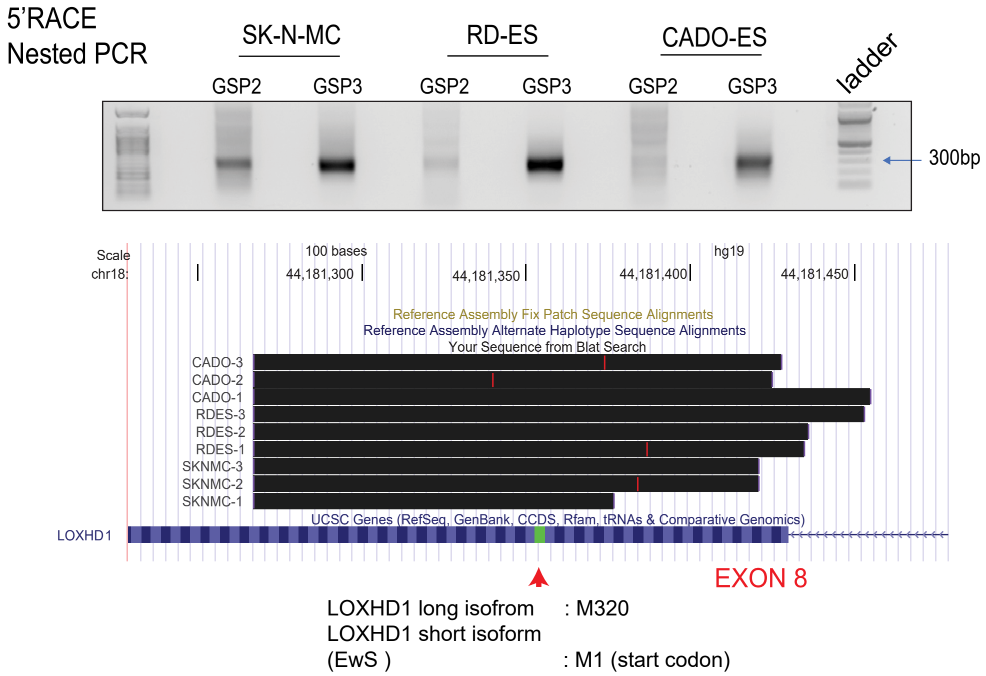

C

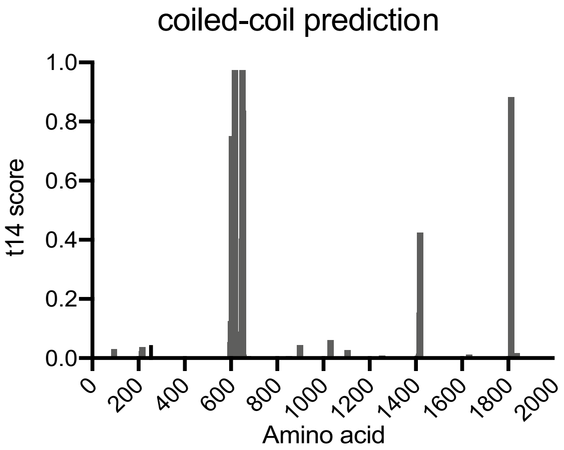

D

Predicted monopartite NLS		
**Pos.**	**Sequence**	**Score**
616	LQRKKKKRKGSDE	15
617	QRKKKKRKGS	6
618	RKKKKRKGSD	7
618	RKKKKRKGSDE	6
1741	IPLKRKRKYFKVF	6
1741	IPLKRKRKY	7
1742	PLKRKRKYFKV	8.5
1743	LKRKRKYFKVF	8.5

E

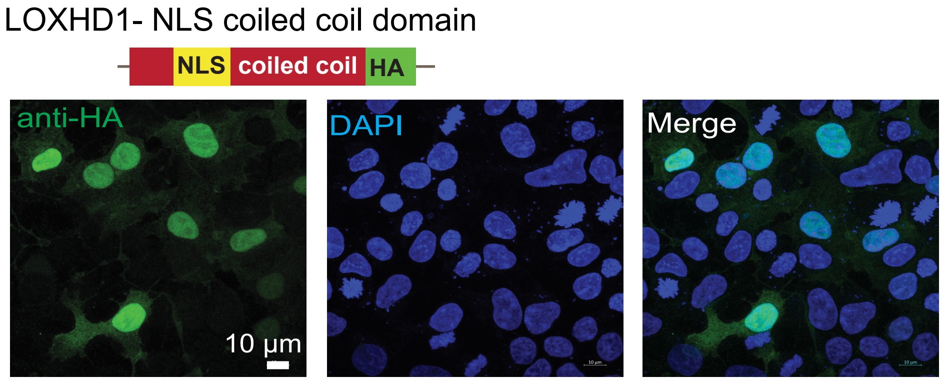

Figure S4: Expression of LOXHD1 short isoform in EwS cell liens, 5' RACE, domain prediction and validation of newly identified NLS (Related to Figure 2).

(A) *top:* Heatmap showing the mRNA FPKM expression in 12 Ewing Sarcoma cell lines. *bottom:* Sashimi plots of aligned RNA-seq data for the mentioned cell lines show the coverage and splice junctions for LOXHD1 transcript. The first seven exons are minimally represented or completely absent in all samples. Arrow indicates exon 8. **(B)** 5' RACE defining the TSS for *LOXHD1* short isoform. *Upper:* 5' RACE using *LOXHD1* gene specific primers (GSP2 and GSP3) in the three indicated cell lines. Agarose gel image showing specific amplicon around 300bp in all three lines in the nested PCR. *Lower:* The sequences from nested PCR products align to the exon 8 of *LOXHD1*. Shown is the screen shot of the UCSC genome browser of the blat result of three independent clones for each cell line. M320 indicated by the red arrowhead could function as the start codon for the LOXHD1 short isoform. **(C)** Coiled-coil structure prediction by COILS (https://embnet.vital-it.ch/software/COILS_form.html), y-axis shows the probability of a 14 amino acid (aa) coiled-coil structure and the x-axis shows the amino acids position of LOXHD1 protein. The predicted coil-coil structure is seen as the peak between aa 653 to aa 677. **(D)** Nuclear localization signals (NLS) predicted by cNLS Mapper (http://nls-mapper.iab.keio.ac.jp/cgi-bin/NLS_Mapper_form.cgi), NLS with a score larger than 8 is exclusively localized to the nucleus. **(E)** Functional validation of NLS. Immunofluorescence staining with HA antibody showing nuclear signal in HEK293T cells transfected with plasmid containing HA-tagged NLS-coiled-coil domain of LOXHD1.

Figure S5

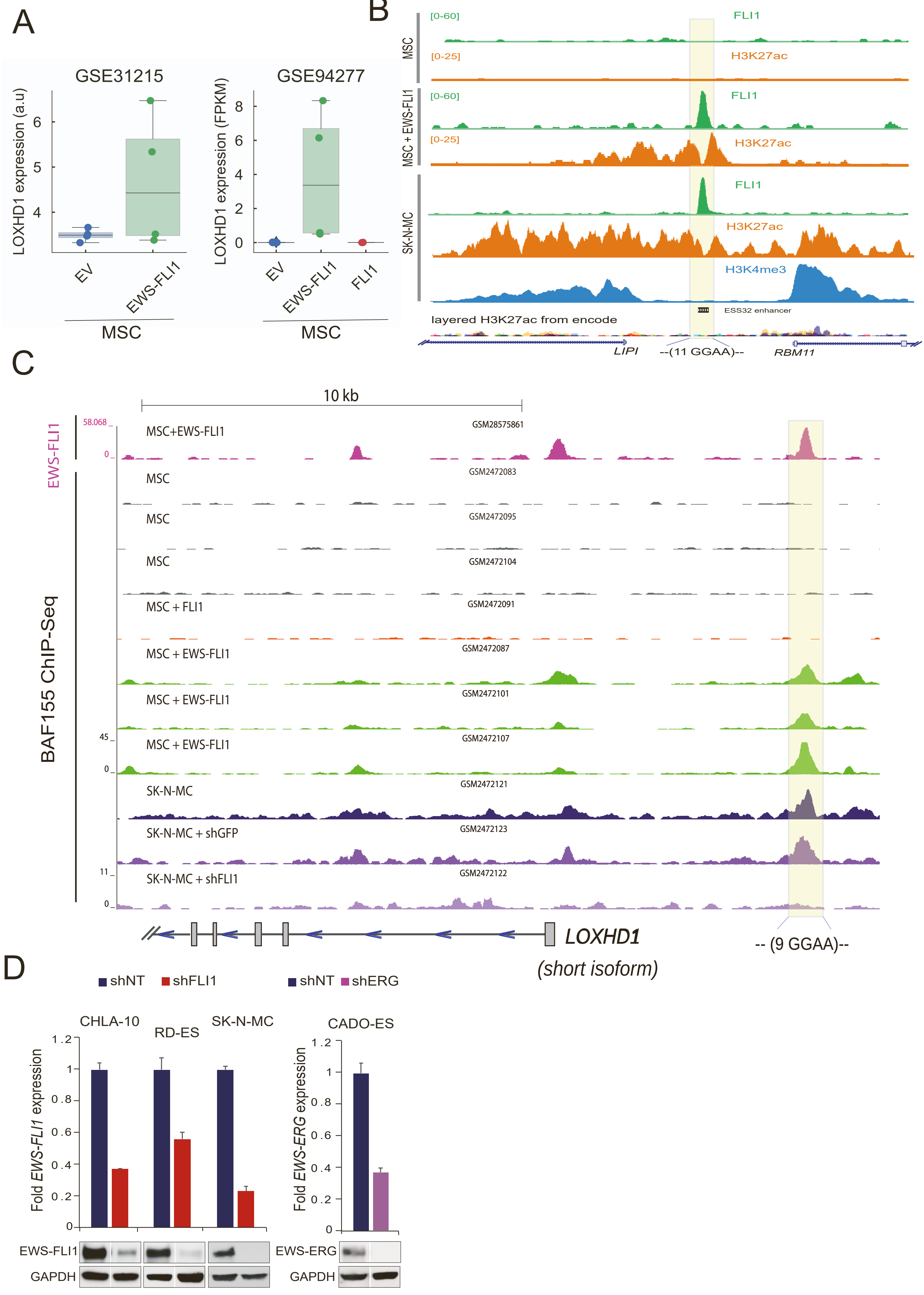
A
GSE31215
LOXHD1 expression (a.u)
EV
EWS-FLI1
MSC
GSE94277
LOXHD1 expression (FPKM)
EV
EWS-FLI1
FLI1
MSC
B
hg19, chr21:15,577,038-15,592,092
5 kb
MSC
MSC + EWS-FLI1
SK-N-MC
[0-60]
FLI1
[0-25]
H3K27ac
H3K4me3
ESS32 enhancer
layered H3K27ac from encode
LIPI
--(11 GGAA)--
RBM11
C
10 kb
EWS-FLI1
BAF155 ChIP-Seq
MSC+EWS-FLI1
GSM28575861
MSC
GSM2472083
MSC
GSM2472095
MSC
GSM2472104
MSC + FLI1
GSM2472091
MSC + EWS-FLI1
GSM2472087
MSC + EWS-FLI1
GSM2472101
MSC + EWS-FLI1
GSM2472107
SK-N-MC
GSM2472121
SK-N-MC + shGFP
GSM2472123
SK-N-MC + shFLI1
GSM2472122
LOXHD1
(short isoform)
-- (9 GGAA)--
D
shNT
shFLI1
shNT
shERG
CHLA-10
RD-ES
SK-N-MC
CADO-ES
Fold EWS-FLI1 expression
Fold EWS-ERG expression
EWS-FLI1
GAPDH
EWS-ERG
GAPDH

Figure S5: *LOXHD1* expression in EWSR1-FLI1 overexpressing MSCs and EWS-FLI knockdown EwS cell lines (Related to Figure 3).

(A) *(left)* Comparison of *LOXHD1* expression profiled using Affymetrix Human Genome U133 Plus 2.0 Array (GSE31215) in MSCs expressing EWSR1::FLI1 (n=4), *(right)* Comparison of *LOXHD1* FPKM expression in MSCs expressing EWSR1::FLI1 (n=4) and wild type FLI1 (n=4), profiled using RNA-seq (GSE94277). **(B)** Genome browser view showing the de novo enhancer assembly in the vicinity of *LIPI* and *RBM11* locus in EWSR1-FLI1 overexpressing MSCs and SK-N-MC cells. **(C)** Genome browser view showing the recruitment of BAF155 (GSE94278) to the LOXHD1 upstream enhancer in MSC EWS-FLI1 overexpressing cells, and loss of BAF155 is apparent in SK-N-MC upon EWS-FLI1 knockdown. **(D)** Bar graph showing qRT-PCR results of FLI1 (*left*) and ERG (*right*) expression with shRNA knockdown of EWSR1::FLI1 and EWSR1::ERG respectively in the indicated EwS cells (n =3, technical replicates). Immunoblot validation for EWSR1::FLI1 and EWSR1::ERG is below. GAPDH was as loading control.

Figure S6

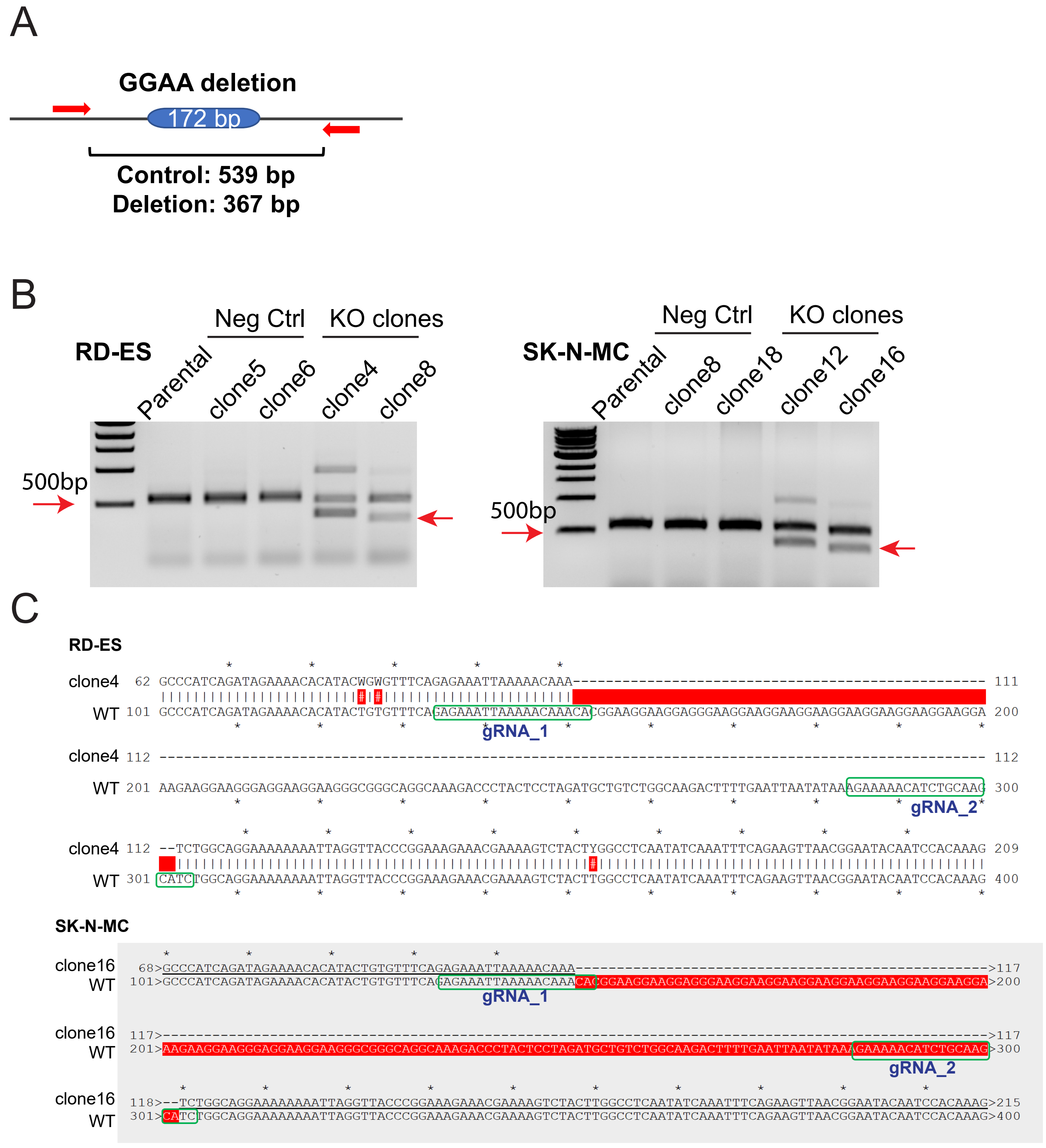

A
GGAA deletion
172 bp
Control: 539 bp
Deletion: 367 bp
B
RD-ES
Neg Ctrl
KO clones
Parental
clone5
clone6
clone4
clone8
500bp
SK-N-MC
Neg Ctrl
KO clones
Parental
clone8
clone18
clone12
clone16
500bp
C
RD-ES
clone4 62 GCCCATCAGATAGAAAACACATACWGWGTTTCAGAGAAATTAAAAACAAA--- 111
WT 101 GCCCATCAGATAGAAAACACATACTGTGTTTCAGAGAAATTAAAAACAAACACGGAAGGAAGGAGGGAAGGAAGGAAGGAAGGAAGGAAGGAAGGAAGGA 200
gRNA_1
clone4 112 --- 112
WT 201 AAGAAGGAAGGGAGGAAGGAAGGGCGGGCAGGCAAAGACCCTACTCCTAGATGCTGTCTGGCAAGACTTTTGAATTAATATAAAGAAAAACATCTGCAAG 300
gRNA_2
clone4 112 --TCTGGCAGGAAAAAAAATTAGGTTACCCGGAAAGAAACGAAAAGTCTACTYGGCCTCAATATCAAATTTCAGAAGTTAACGGAATACAATCCACAAAG 209
WT 301 CATCTGGCAGGAAAAAAAATTAGGTTACCCGGAAAGAAACGAAAAGTCTACTTGGCCTCAATATCAAATTTCAGAAGTTAACGGAATACAATCCACAAAG 400
SK-N-MC
clone16 68>GCCCATCAGATAGAAAACACATACTGTGTTTCAGAGAAATTAAAAACAAA--->117
WT 101>GCCCATCAGATAGAAAACACATACTGTGTTTCAGAGAAATTAAAAACAAACACGGAAGGAAGGAGGGAAGGAAGGAAGGAAGGAAGGAAGGAAGGAAGGA>200
gRNA_1
clone16 117>--->117
WT 201>AAGAAGGAAGGGAGGAAGGAAGGGCGGGCAGGCAAAGACCCTACTCCTAGATGCTGTCTGGCAAGACTTTTGAATTAATATAAAGAAAAACATCTGCAAG>300
gRNA_2
clone16 118>--TCTGGCAGGAAAAAAAATTAGGTTACCCGGAAAGAAACGAAAAGTCTACTTGGCCTCAATATCAAATTTCAGAAGTTAACGGAATACAATCCACAAAG>215
WT 301>CATCTGGCAGGAAAAAAAATTAGGTTACCCGGAAAGAAACGAAAAGTCTACTTGGCCTCAATATCAAATTTCAGAAGTTAACGGAATACAATCCACAAAG>400

Figure S6: Verifications of the LOXHD1 enhancer knockout single clones (Related to Figure 4).

(A) PCR and sequencing strategy for GGAA microsatellite deletion. Primers designed outside of the two gRNAs flanking the GGAA microsatellite; wild-type allele predicted to have 539bp amplicon while the deleted allele is predicted to have 367bp amplicon. **(B)** DNA gel image of PCR products with the genomic DNA of empty cas9 control cells and the enhancer targeting sgRNA CRISPR-cas9 virus infected cells. SK-N-MC and RD-ES cells were transduced with empty gRNA cas9 virus or cas9 with two gRNAs flanking the GGAA microsatellite, three days post viral transduction cells were subjected for puromycin selection for three days and harvested for genomic DNA for PCR. Arrow indicates the allele with 172bp deletion. **(C)** Sanger sequencing result of the enhancer knockout RD-ES and SK-N-MC single cell derived clones. Red region shows the deletion between two guide RNAs.

Figure S7

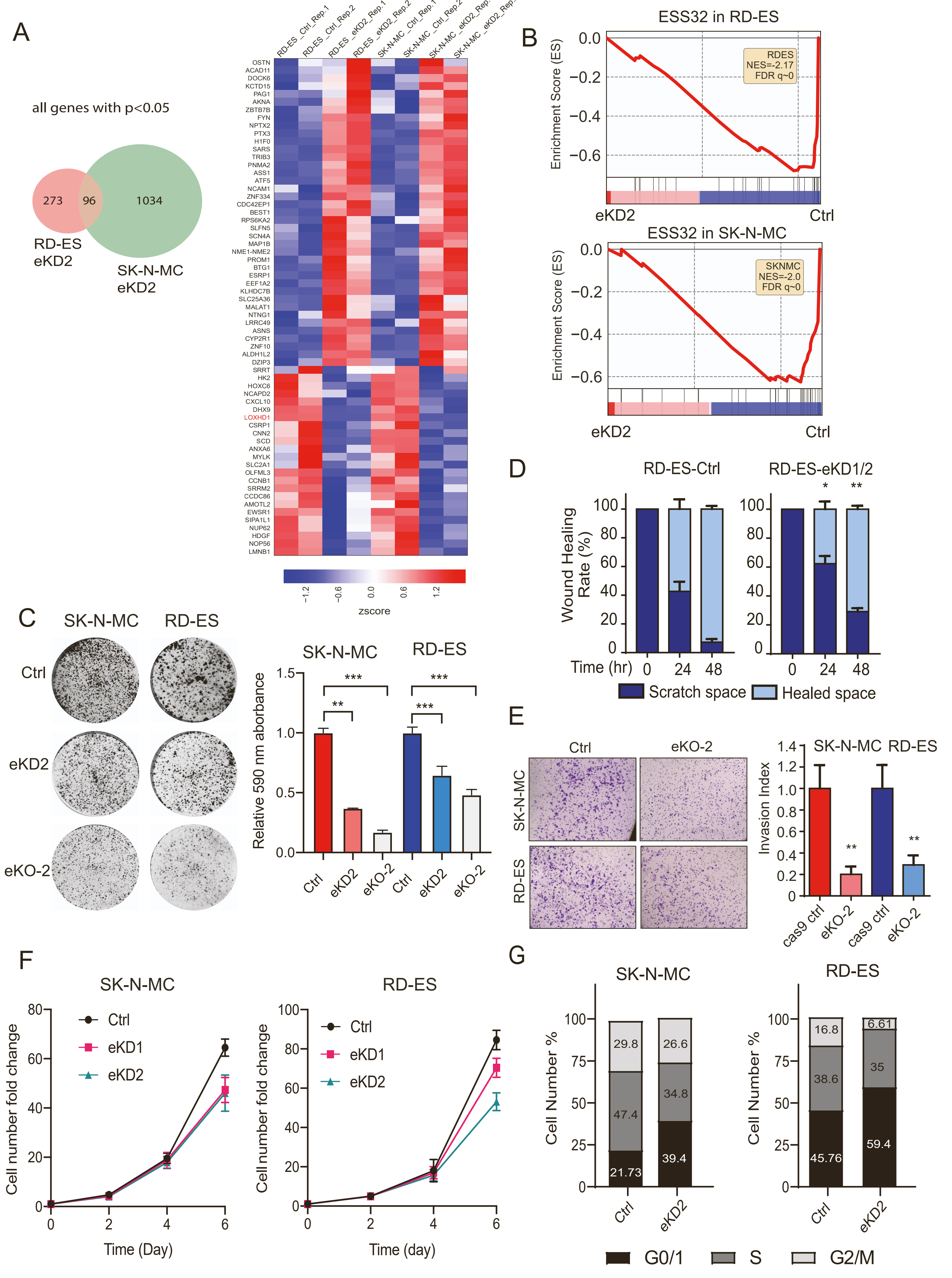

A
all genes with p<0.05
273
96
1034
RD-ES eKD2
SK-N-MC eKD2
zscore
B
ESS32 in RD-ES
RDES NES=-2.17 FDR q~0
ESS32 in SK-N-MC
SKNMC NES=-2.0 FDR q~0
Enrichment Score (ES)
eKD2
Ctrl
C
SK-N-MC
RD-ES
Ctrl
eKD2
eKO-2
Relative 590 nm aborbance
D
RD-ES-Ctrl
RD-ES-eKD1/2
Wound Healing Rate (%)
Time (hr)
Scratch space
Healed space
E
Ctrl
eKO-2
Invasion Index
cas9 ctrl
F
SK-N-MC
RD-ES
Cell number fold change
eKD1
Time (Day)
Time (day)
G
Cell Number %
G0/1
S
G2/M

Figure S7: LOXHD1 loss impairs EwS signature and tumor phenotype *in vitro* (Related to Figure 5).

(A) Venn diagram and heatmap showing differentially expressed genes in LOXHD1 enhancer knockdown (eKD2) cells. n=2 biological replicates. **(B)** RNA-seq followed by GSEA showing negative enrichment of the ESS32 signature in *LOXHD1* eKD2 RD-ES and SK-N-MC cells. **(C)** LOXHD1 knockdown impairs colony formation ability in EwS cells. *left* Representative images of a colony formation assay performed by seeding 5000 control, eKD and eKO cells in each well of 6-well plates, n=3 technical replicates. *right*, bar graph showing quantifications of the crystal violet staining of the colonies. LOXHD1 knockdown slows down cell migration in EwS cells. **(D)** Bar graph of a wound healing assay presented by quantifications of the scratch widths in RD-ES control and eKD cells. LOXHD1 knockdown reduces cell invasion in EwS cells. **(E)** *left*, representative images of a Boyden chamber invasion assay with SK-N-MC and RD-ES control and eKO single cell clones. *right*, bar graph showing quantifications of the invaded cells. (**F**) Growth curve of SK-N-MC, RD-ES control and two eKD cells grown in the regular culture condition. Cell numbers were counted and relative fold increase plotted with mean±S.D from n=3. **(G)** LOXHD1 silencing stalls the cells to the G0/1 phase. The indicated cells cultured for five days were trypsinized and stained with Propidium Iodide for cell cycle analysis by flow cytometry. The percentage of each cell cycle phase is indicated in the graph. Experiments of panel C-G were performed ≥ 3 times.

Figure S8

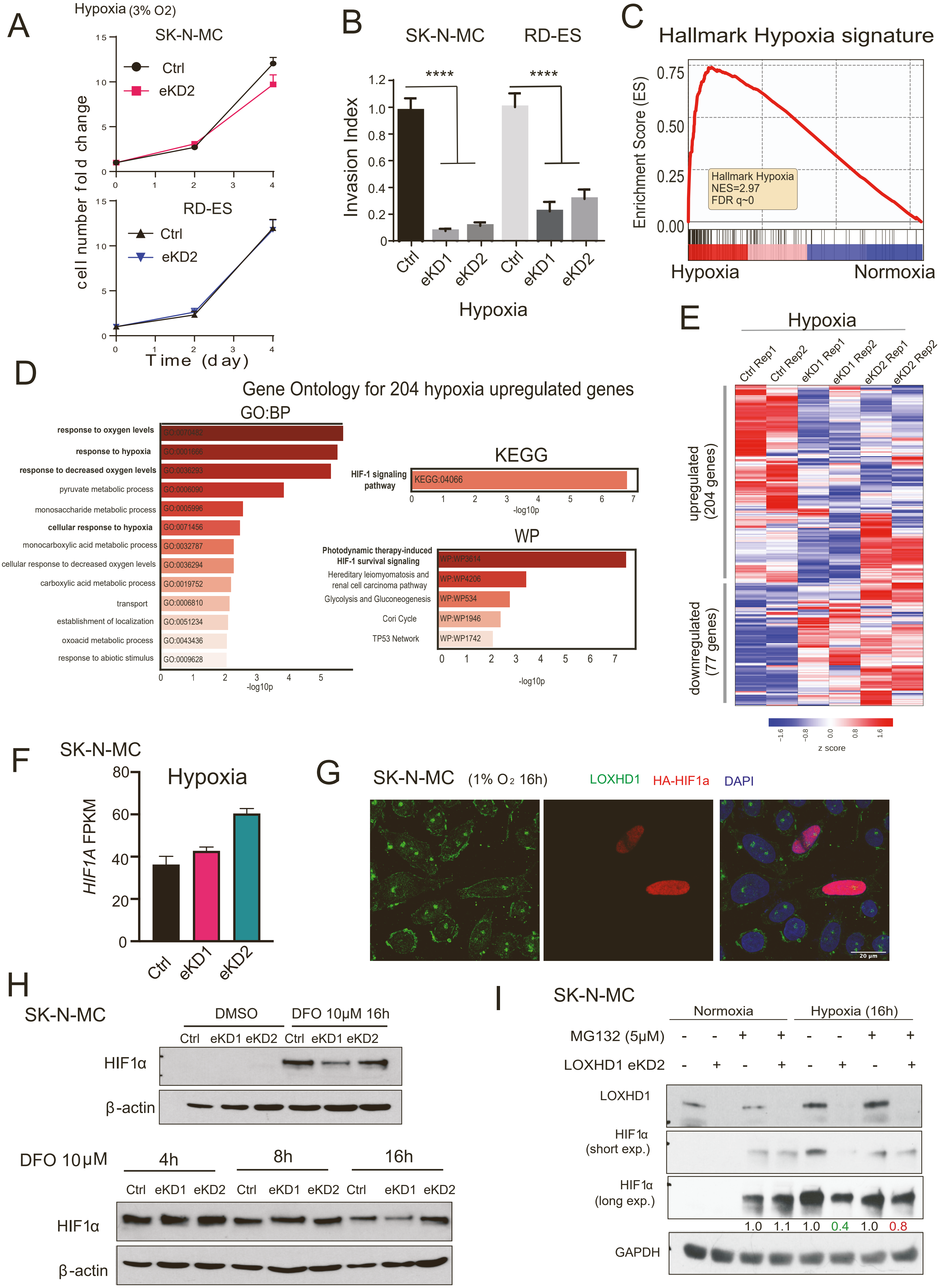

A
Hypoxia (3% O2)
SK-N-MC
Ctrl
eKD2
RD-ES
Ctrl
eKD2
cell number fold change
Time (day)
B
SK-N-MC
RD-ES
Invasion Index
Ctrl
eKD1
eKD2
Hypoxia
C
Hallmark Hypoxia signature
Enrichment Score (ES)
Hallmark Hypoxia
NES=2.97
FDR q~0
Hypoxia
Normoxia
D
Gene Ontology for 204 hypoxia upregulated genes
GO:BP
response to oxygen levels
response to hypoxia
response to decreased oxygen levels
pyruvate metabolic process
monosaccharide metabolic process
cellular response to hypoxia
monocarboxylic acid metabolic process
cellular response to decreased oxygen levels
carboxylic acid metabolic process
transport
establishment of localization
oxoacid metabolic process
response to abiotic stimulus
-log10p
KEGG
HIF-1 signaling pathway
KEGG:04066
WP
Photodynamic therapy-induced HIF-1 survival signaling
Hereditary leiomyomatosis and renal cell carcinoma pathway
Glycolysis and Gluconeogenesis
Cori Cycle
TP53 Network
E
Hypoxia
Ctrl Rep1
Ctrl Rep2
eKD1 Rep1
eKD1 Rep2
eKD2 Rep1
eKD2 Rep2
upregulated (204 genes)
downregulated (77 genes)
z score
F
SK-N-MC
Hypoxia
HIF1A FPKM
G
SK-N-MC (1% O₂ 16h)
LOXHD1
HA-HIF1a
DAPI
20 μm
H
SK-N-MC
DMSO
DFO 10μM 16h
HIF1α
β-actin
DFO 10μM
4h
8h
16h
I
SK-N-MC
Normoxia
Hypoxia (16h)
MG132 (5μM)
LOXHD1 eKD2
LOXHD1
HIF1α (short exp.)
HIF1α (long exp.)
1.0 1.1 1.0 0.4 1.0 0.8
GAPDH

Figure S8: LOXHD1 knockdown diminishes hypoxic responses in EwS cells (Related to Figure 6).

Hypoxia enhances the effects of LOXHD1 knockdown on EwS cell invasion. **(A)** Growth curve of SK-N-MC, RD-ES control eKD2 cells under hypoxia (3% oxygen). **(B)** Bar graph showing quantifications of invasion index of the hypoxia samples in the experiment of Fig. 6A. LOXHD1 proficient SK-N-MC cells present strong hypoxia response. n=3 technical replicates, experiments in panel A-B were performed three times with similar results. (**C)** RNA-seq followed by GSEA showing a positive enrichment of Hallmark Hypoxia signature in the hypoxic treated control SK-N-MC cells compared to the normoxia sample. **(D)** Gene Ontology for the 204 genes induces by hypoxia showing hypoxic responses and HIF-1signaling among the top of the lists. LOXHD1 knockdown in EwS cells does not affect *HIF1A* transcription. (**E**) LOXHD1 silencing reverses hypoxia response in SK-N-MC cells. Heatmap shows hypoxia upregulated and downregulated genes in the indicated cells. **(F)** Bar graph of the FPKM of *HIF1A* in control and two eKD SK-N-MC cells under hypoxia. **(G)** LOXHD1 and HIF1α colocalize in the nucleus. Immunofluorescent staining of the endogenous LOXHD1 and HA-tagged HIF1α showing nuclear co-localized signal in SK-N-MC cells after 16h of hypoxia culture. After fixation, HIF1a was stained using anti-HA ab, nuclei with DAPI. Localizations were observed using confocal laser scanning microscopy. Scale bars, 20 μm. **(H)** Immunoblot of HIF1α with DFO treatment at 10 μM for 4, 8 and 16h in control and two eKD SK-N-MC cells. *top:* DMSO was used as a negative control for the DFO treatment. β actin was used as loading control. **(I)** Immunoblots of LOXHD1 and HIF1α of the indicated cell lines and conditions, MG132 treatment was for 8hr. GAPDH used as a loading control. Normalized quantifications indicated are relative to the control cells.

Figure S9

A

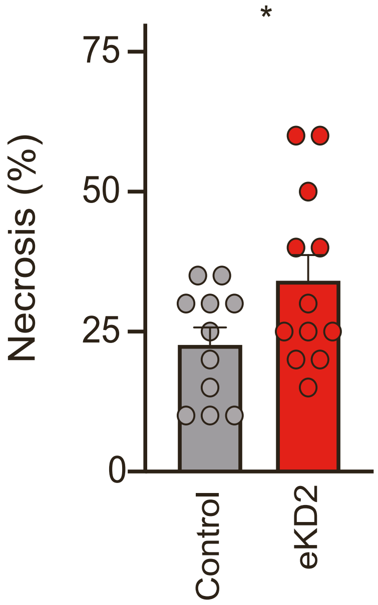

*
75
50
25
0
Necrosis (%)
Control
eKD2

B

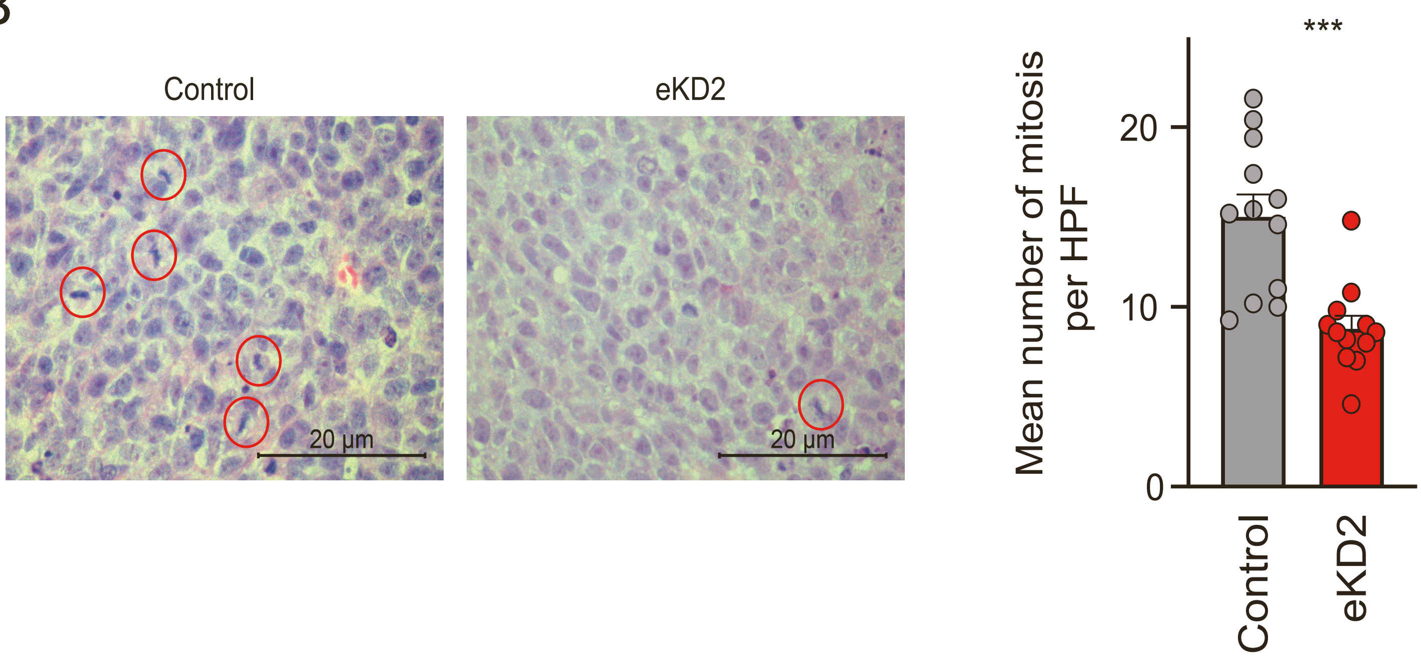

Control
eKD2
20 µm
20 µm
***
20
10
0
Mean number of mitosis per HPF
Control
eKD2

Figure S9: LOXHD1 knockdown affects EwS growth in vivo (Related to Figure 7).

(A) Bar graph showing the percentage of necrotic regions in the control and LOXHD1 enhancer knockdown SK-N-MC xenograft. n=11 for control group, n=12 for eKD2 group. **(B)** Representative images showing mitotic nuclei in the control and LOHXD1 enhancer knockdown xenograft. The bar graph show average mitotic nuclei of all the tumors analyzed.

Table S1: Information on the total number of samples including EwS in five curated RNA-seq datasets. **(Related to Figure 1)**

Dataset	# samples	# EwS samples
MET500+EwS	507	11
CCLE	980	9
GTEX (v6p)	11401	0
TCGA (12/10/2019)	9205	0
DepMap	1376	22

Table S2: GEO accession IDs and corresponding cell lines for RNA-seq and ChIP-seq data used in various figures. (Related to Figures 1-3)

Figure	#datasets	cell lines	GEO Accession Ids
Fig 1A: step1 RNA-seq	3	A673, SK-N-MC, CHLA	GSE61953, GSE113604
Fig1A: step3 FLI1 ChIP-seq	3	A673, SK-N-MC, MSC	GSE61953, GSE94275
Fig1B	5	A673, SK-N-MC, MSC	GSE45414, GSE94277, GSE113604, GSE31215)
Fig 1B, right panel	3	A673, SK-N-MC, MSC	GSE61953, GSE94275
Fig. 1C	9	A673, SK-N-MC, MSC, primary EwS tissues	GSE61944
Fig. 2A	12	12 different EwS lines	GSE98787, GSE119970, GSE124221, GSE113604
Fig. 3A	7	MSC, SK-N-MC	GSE94278, GSE106914, GSE61953
Fig, 3C	10	SK-N-MC, A673	GSE61944

Table S3. Details on ESS32 genes (Related to Figure 1)

#	ESS32 Gene list	Full name	Gene ontology (biological process)	Function	Status in Ewing sarcoma literature		
1	KLF15	Kruppel Like Factor 15	positive regulation of transcription by RNA polymerase II [GO:0045944]	Encodes for a transcriptional regulator that binds to, among other promoter regions, the CLCNKA promoter. It is known to inhibit MEF2A and GATA4, thereby playing a role in controlling cardiac hypertrophy. It has also been elucidated as a negative regulator of TP53 acetylation.	Todd M. Stevens, International Journal of Surgical Pathology, 2018		
2	PDE1B	Phosphodiesterase 1B	apoptotic process [GO:0006915]	Cyclic nucleotide phosphodiesterase with a dual-specificity for the second messengers cAMP and cGMP, which are key regulators of many important physiological processes. Has a preference for cGMP as a substrate	None		
3	STEAP2	STEAP2 Metalloreductase	copper ion import [GO:0015677]	Metalloreductase that has the ability to reduce both Fe(3+) to Fe(2+) and Cu(2+) to Cu(1+).	Inês M. Gomes, Molecular Cancer Research, 2012	Thomas G. P. Grunewald, Biology of the Cell, 2012	
4	ABI3	ABI Family Member 3	regulation of cell migration [GO:0030334]	The encoded protein is known to inhibit ectopic metastasis of tumor cells and cell migration via interaction with p21-activated kinase.	None		
5	DNAJC12	DnaJ Heat Shock Protein Family (Hsp40) Member C12	None	Encodes for a member of a subclass of the HSP40/DnaJ protein family, which are known to associate with complex assembly, protein folding, and export.	None		
6	PPP1R1A	Protein Phosphatase 1 Regulatory Inhibitor Subunit 1A	intracellular signal transduction [GO:0035556]	Inhibitor of protein-phosphatase 1	Wen Luo, Nature Oncogene, 2018	Wen Luo, Nature Oncotarget, 2020	Daniel H Wai, International Journal of Oncology, 2002
7	NPY1R	Neuropeptide Y Receptor Y1	adenylate cyclase-inhibiting G-protein coupled receptor signaling pathway [GO:0007193]	Receptor for neuropeptide Y and peptide YY.	Jason U. Tilan, Oncotarget, 2013		

8	VAV1	Vav Guanine Nucleotide Exchange Factor 1	regulation of GTPase activity [GO:0043087]	VAV proteins are guanine nucleotide exchange factors (GEFs) for Rho family GTPases, which activate downstream pathways that lead to actin cytoskeletal rearrangements and transcriptional changes.	Rodolphe Guinamard, Scandinavian Journal of Immunology, 1997		
9	DUSP26	Dual Specificity Phosphatase 26	protein dephosphorylation [GO:0006470]	Encodes for a tyrosine phosphatase, and has the ability to dephosphorylate both tyrosine and serine/threonine residues. Protein product may regulate neuronal proliferation. This gene has been described as both a tumor suppressor and an oncogene.	None		
10	KCNAB3	Potassium Voltage-Gated Channel Subfamily A Regulatory Beta Subunit 3	ion transmembrane transport [GO:0034220]	Encodes for protein that forms a heterodimer with the potassium voltage-gated channel, shaker-related subfamily of proteins.	None		
11	RBM11	RNA Binding Motif Protein 11	cell differentiation [GO:0030154]	Tissue-specific splicing factor with potential implication in the regulation of alternative splicing during neuron and germ cell differentiation.	Andrew J. Annalora, Oncotarget, 2018		
12	KCNE3	Potassium Voltage-Gated Channel Subfamily E Regulatory Subunit 3	negative regulation of voltage-gated potassium channel activity [GO:1903817]	Regulates neurotransmitter release, heart rate, insulin secretion, neuronal excitability, epithelial electrolyte transport, smooth muscle contraction, and cell volume.	None		
13	MYOM2	Myomesin 2	muscle contraction [GO:0006936]	Binds to myosin, titin, and light meromyosin. Shares genetic homology to fibronectin type III and immunoglobulin C2 domains.	None		
14	PRR5L	Proline Rich 5 Like	negative regulation of protein phosphorylation [GO:0001933]	Associates with the mTORC2 complex, thereby regulating cellular processes such as survival and cytoskeletal organization.	None		
15	NKX2-2	NK2 Homeobox 2	positive regulation of transcription by RNA polymerase II [GO:0045944]	Protein-coding gene that contains a homeobox domain and has possible role in the morphogenesis of the central nervous system.	Mitchel J. Machiela, Nature Communications, 2018	Leah A. Owen, PLoS One, 2008	Richard Smith, Cancer Cell, 2006
16	XG	Xg Glycoprotein	homotypic cell-cell adhesion [GO:0034109]	Encodes for the XG blood group antigen	ynet, Cancer Research, 2010		

17	RNF182	Ring Finger Protein 182	protein ubiquitination [GO:0016567]	Encodes for E3 ubiquitin-protein ligase. Mediates the ubiquitination of ATP6V0C and marks it for degradation through the ubiquitin-proteasome pathway. Inhibits the TLR triggered innate immune response via ubiquitination and subsequent degradation of NF-kappa-B component RELA.	None		
18	KCNA2	Potassium Voltage-Gated Channel Subfamily A Member 2	potassium ion transport [GO:0006813]	A voltage-gated potassium channel found primarily in the brain, central nervous system, and the cardiovascular system.	None		
19	PRRT4	Proline Rich Transmembrane Protein 4	None	A protein-coding gene associated with the disease, Zellweger Syndrome.	None		
20	NPY5R	Neuropeptide Y Receptor Y5	cardiac left ventricle morphogenesis [GO:0003214]	Receptor for neuropeptide Y and peptide YY.	Jason U. Tilan, Oncotarget, 2013	Jason Tilan, Neuropeptides, 2016	Congyi Lu, The Journal of Biological Chemistry, 2011
21	TNNI3	Troponin I3, Cardiac Type	muscle contraction [GO:0006936]	Part of the Troponin I subfamily of genes. It encodes for the TnI-cardiac protein which is only expressed in cardiac muscle tissues.	None		
22	ARTN	Artemin	neuroblast proliferation [GO:0007405]	Encodes for the ligand that activates the GFR-alpha-3-RET receptor complex.	None		
23	CD79A	CD7B-Cell Antigen Receptor Complex-Associated Protein Alpha Chain9a	B cell receptor signaling pathway [GO:0050853]	A B lymphocyte antigen receptor that works in conjunction with CD79B to initiate the signal transduction cascade activated by the binding of an antigen to the B-cell antigen receptor complex.	David R. Lucas, Anatomic Pathology, 2001	Metin Ozdemirli, Nature Modern Pathology, 2001	Metin Ozdemirli, The American Journal of Surgical Pathology,
24	PHOSPHO 1	Phosphoethanolamine/Phosphocholine Phosphatase 1	bone mineralization involved in bone maturation [GO:0035630]	A phosphatase that has a high specificity for phosphoethanolamine (PEA) and phosphocholine (PCho). Plays a role in generating inorganic phosphate for bone mineralization.	None		
25	ADRB3	Adrenoceptor Beta 3	adenylate cyclase-modulating G protein-coupled receptor signaling pathway [GO:0007188]	The protein product is part of the beta adrenergic receptor family, and is involved in the regulation of lipolysis and thermogenesis.	Andreas Kirschner, Oncotarget, 2016		

26	DCDC2	Doublecortin Domain Containing 2	cellular defense response [GO:0006968]	Encodes for protein that plays a role in the inhibition of canonical Wnt signaling pathway.	None		
27	GNGT2	G Protein Subunit Gamma Transducin 2	G protein-coupled receptor signaling pathway [GO:0007186]	Encodes for protein that plays a crucial role in cone phototransduction, and is specifically localized in cones.	None		
28	FEZF1	FEZ Family Zinc Finger 1	neuron migration [GO:0001764]	Encodes for a transcriptional repressor protein, and is involved in the axonal projection and proper termination of olfactory sensory neurons.	None		
29	UGT3A2	UDP Glycosyltransferase Family 3 Member A2	cellular response to genistein [GO:0071412]	UDP-glucuronosyltransferases catalyze phase II biotransformation reactions in which lipophilic substrates are conjugated with glucuronic acid to increase water solubility and enhance excretion.	None		
30	LOXHD1	Lipoxygenase Homology Domain-Containing Protein 1	calcium ion transmembrane transport [GO:0070588]	Involved in hearing. Required for normal function of hair cells in the inner ear.	None		
31	LIPI	Lipase I	a potent bioactive lipid mediator) and fatty acid. Does not hydrolyze other phospholipids, like phosphatidylserine (PS), phosphatidylcholine (PC) and phosphatidylethanolamine (PE) or triacylglycerol (TG). {ECO:0000269|PubMed:12963729}.	Hydrolyzes specifically phosphatidic acid (PA) to produce 2-acyl lysophosphatidic acid (LPA	Juergen L. Foell, Pediatric Blood & Cancer, 2008	Dorothea E. Mahlendorf, Cancer Biology & Therapy, 2013	Benjamin J. Schmiedel, Molecular Biology Reports, 2011
32	RAX	Retina And Anterior Neural Fold Homeobo	visual perception [GO:0007601]	Contains a homeobox domain, and encodes for a transcription factor known to have functions in eye development. Required for retinal cell fate determination and regulates stem cell proliferation.	None		

Table S4: Overlap of ESS32 with published EWS-ETS driven EwS specific signature (Related to Figure 1)

**Dataset**	**#genes**	**#overlap with ESS32**	**overlapping genes**	**Reference**
IC10	**220**	**9**	DNAJC12, DCDC2, STEAP2, NPY1R, NPY5R, LOXHD1, UGT3A2, RBM11, LIPI	Riggi N et. Al., Cancer Cell, 2014
EWS78	**78**	**4**	DCDC2, UGT3A2, RBM11, LIPI	Aynaud, M et. al., Cell Rep., 2020
EW446	**446**	**9**	NKX2-2, DUSP26, PPP1R1A, STEAP2, NPY1R, KCNE3, RNF182, RBM11, ADRB3	Gordon, DJ et. al., Oncogene, 2015
EW33 (non survivors vs survivors)	**33**	**0**	-	Volchenboum, SL et al, J Pathol Clin Res, 2015

Table S6: List of Oligonucleotide Primers used in this study (Related to STAR methods)

**CRISPR gRNAs**	**Sequences 5' – 3'**
GGAA Enhancer KO gRNA_1 FWD	CACCGAGAGAAATTAAAAACAAACA
GGAA Enhancer KO gRNA_1 REV	AAACTGTTTGTTTTTAATTTCTCTC
GGAA Enhancer KO gRNA_2 FWD	CACCGGAAAAACATCTGCAAGCATC
GGAA Enhancer KO gRNA_2 REV	AAACGATGCTTGCAGATGTTTTTCC
GGAA dcas9 gRNA_1 FWD	CACCGAGAGAAATTAAAAACAAACA
GGAA dcas9 gRNA_1 REV	AAACTGTTTGTTTTTAATTTCTCTC
GGAA dcas9 gRNA_2	CACCGGTAGAGATGACAGGAGTAAA
GGAA dcas9 gRNA_2	AAACTTTACTCCTGTCATCTCTACC
**Cloning**	
pcDNA3-LOXHD1-Myc NG-138	ATGATGGCCCAGAAGAAGAAGCGGAG
pcDNA3-LOXHD1-Myc NG-64	ACACCCTGCAGCAAGTCCCAACC
pcDNA3-LOXHD1-Myc PW-83	GAACAAAAACTCATCTCAGAAGAGGATCTTGAGAATT CCACCACACTG
pcDNA3-LOXHD1-Myc PW-84	TCTGAGATGAGTTTTTGTTCAACGGCCGCGACAGACGG GAAGAGCTC
NLS-coiled-coil-HA-NG-191-NheI-CC-S	CAGCTGGCTAGCACCATGGTGTGGCTGC GGCACCTGGTG
NLS-coiled-coil-HA-NG-192-NotI-CC-AS	TTGCGGCCGCTCAAGCGTAATCTGGAACATCGTA TGGGTAAACGGCCGCAATCACCTCCTGCATCCCT GGCC
**Genomic PCR validation for enhancer deletion**	
GGAA enhancer FWD	AAGTGGAACTCAGTGTGGAACA
GGAA enhancer REV	GCAGGGCACAGAACAGGTACCT
**SYBR Green qPCR**	
LOXHD1 FWD	TAGTGACCAGGCTGGGACTTG
LOXHD1 REV	GCTTCTCCACTTCTATCCCCT

FLI-1 FWD	TTAAGGAGGCTCTGTCGGTG
FLI-1 REV	GAGGGGGTTGATCTTGTGGG
CCK FWD	AGGGTATCGCAGAGAACGGA
CCK REV	GGGCCTGCTGGATGTATCTT
GAPDH FWD	TGCACCACCAACTGCTTAGC
GAPDH REV	GGCATGGACTGTGGTCATGAG
**ChIP qPCR**	
GGAA microsatellite FWD	AAACAAATAGCCTGCCCATCAG
GGAA microsatellite REV	CCCTCCTTCCTTCCGTGTTT
LOXHD1 TSS FWD	CTCAGGTTCCCGCAGGTGT
LOXHD1 TSS REV	GGGGCATCATTCTGTCGGC
Non-Specific control FWD	ATCCCCCACAACTCCACCTA
Non-Specific control REV	ACAGGTAGCAACGAACTGGG
**5' RACE**	
GSP1	AGCCAGTTGCTACAAGGGAA
GSP2	TGTTGACACCCACATTGCCA
GSP3	ATGTGGAAGATGTCCGTGCG
**Human Alu Taqman qPCR**	
Human Alu FWD	GTCAGGAGATCGAGACCATCCT
Human Alu REV	AGTGGCGCAATCTCGGC
Human Alu Taqman probe	5′-6-FAM-AGCTACTCGGGAGGCTGAGGCAGGA-TAMRA-3′
